# Metabolic syndrome components individually worsen the outcome of acute pancreatitis: a systematic review and meta-analysis

**DOI:** 10.3389/fendo.2025.1690754

**Published:** 2025-11-04

**Authors:** Dalma Dobszai, Mahmoud Obeidat, Eszter Ágnes Szalai, Dániel Sándor Veres, Luca Havelda, Renáta Papp, Alim Choi, Bettina Csilla Budai, Dominika Csajbók, Péter Hegyi, Andrea Szentesi

**Affiliations:** ^1^ Institute for Translational Medicine, Medical School, University of Pécs, Pécs, Hungary; ^2^ Centre for Translational Medicine, Semmelweis University, Budapest, Hungary; ^3^ Department of Restorative Dentistry and Endodontics, Semmelweis University, Budapest, Hungary; ^4^ Department of Biophysics and Radiation Biology, Semmelweis University, Budapest, Hungary; ^5^ Institute of Pancreatic Diseases, Semmelweis University, Budapest, Hungary; ^6^ Department of Pharmacology and Pharmacotherapy, Semmelweis University, Budapest, Hungary; ^7^ Center for Pharmacology and Drug Research and Development, Semmelweis University, Budapest, Hungary; ^8^ Translational Pancreatology Research Group, Interdisciplinary Centre of Excellence for Research Development and Innovation, University of Szeged, Szeged, Hungary

**Keywords:** acute, pancreatitis, metabolic, syndrome, obesity, diabetes, hypertriglyceridemia, hypertension

## Abstract

**Introduction:**

The obesity epidemic has led to a rise in related health conditions, with metabolic syndrome (MS) affecting 25% of Western populations. In severe acute pancreatitis (AP), mortality can reach 50%. Previous studies have linked MS elements to increased complications and mortality in AP. This meta-analysis aims to identify potential risk factors and their synergistic effects on AP outcomes.

**Methods:**

We systematically searched PubMed, Embase, and the Cochrane Library up to November 1, 2023, and included studies based on predefined criteria. We examined the impact of MS and its factors (obesity, hypertension, diabetes mellitus, and hypertriglyceridemia) on AP outcomes, calculating pooled odds ratios (OR) with 95% confidence intervals (CIs). The protocol was registered in PROSPERO under number CRD42023471092.

**Results:**

Out of 15,904 records, 89 studies were analyzed. Overweight and obesity were significant risk factors for complications (local OR: 2.677, 95%CI: 1.421-5.044; systemic OR: 2.404, 95%CI: 1.481-3.901) and severe AP (BMI≥30 kg/m^2^, OR: 3.058, 95%CI: 1.369-6.829). High triglyceride levels were associated with ICU admission (OR: 2.546, 95%CI: 1.529-4.237) and severe AP (OR: 2.686, 95%CI: 1.205-5.989); hypertension increased mortality (OR: 2.135, 95%CI: 1.870-2.437), while diabetes increased the odds of ICU admission (OR: 1.645, 95%CI: 1.358-1.992) and severe AP (OR: 1.49, 95%CI: 1.09-2.03). We found a non-significant trend toward increased odds of severe AP among patients with MS (OR = 1.398, 95% CI: 0.918–2.129).

**Conclusion:**

Individual components of MS are risk factors for complications, severity, and mortality in AP. Lifestyle counseling, education, and treatment of patients with obesity is crucial.

**Systematic Review Registration:**

https://www.crd.york.ac.uk/prospero/, identifier CD42023471092.

## 1 Introduction

Acute pancreatitis (AP) is a potentially life-threatening gastrointestinal condition requiring hospitalization, with its overall incidence increasing by 3% annually over the past 50 years, placing a huge burden on national healthcare systems (1). While improvements in diagnostic tools and criteria have contributed to this rise, lifestyle factors such as sedentary behavior, poor nutrition, increased alcohol consumption, medication use, smoking, and socioeconomic status also play a major role (1–3). AP can lead to various local and systemic complications linked to pre-existing conditions, with severe cases resulting in organ failure (4). Patients going through transient organ failure or developing local/systemic complications without organ failure are classified as moderately severe, whereas severe cases are characterized by persistent organ failure (4).

Globally, up to 39% of the population is classified as obese or overweight. Since the 1970s, the prevalence of obesity has risen threefold, making it the fifth leading cause of death worldwide (5). Obesity is associated with a greater risk of inpatient mortality, higher hospitalization costs, and overall poorer clinical outcomes in cases of AP (6). A meta-analysis covering all BMI categories revealed that a BMI≥25 elevates the risk of severe AP without impacting mortality, whereas a BMI greater than 30 is linked to increased mortality risk (7). Additionally, our previous cohort analysis identified obesity as an independent predictor of renal failure and prolonged hospital stay (8).

Obesity significantly increases the risk of various chronic comorbidities (such as type 2 diabetes mellitus, dyslipidemia, hypertension, and cancer) (9) that often correlate with poorer outcomes in acute inflammatory conditions (10–13). In particular, visceral fat accumulation is linked to numerous cardiovascular issues as well as increased fat deposits in the liver, muscle, and pancreas (5).

Metabolic syndrome (MS) covers a range of interrelated metabolic disorders that significantly elevate the risk for cardiovascular diseases and overall mortality (14). While definitions vary (15), it typically involves the presence of at least three of the following four conditions: abdominal obesity, impaired glucose metabolism, hypertension, and dyslipidemia. The incidence of MS has sharply risen in recent decades, raising significant public health concerns worldwide (16). The development of MS is influenced by various factors, with lifestyle choices—particularly poor dietary habits and lack of physical activity—playing a crucial role alongside genetic and environmental influences (17).

Given the potentially fatal nature of AP and its complications, the early identification of patients at high risk of developing severe forms of the disease is critically important. To be able to create preventive treatments for severe AP in the future, there is a desperate need to determine the risk of complications and predict severity using the information available upon admission, but at most within the first 24 hours of hospital admission (18).

Earlier studies have associated the components of MS with a higher chance of developing severe AP and an increased risk of mortality (7, 8, 13), however, no comprehensive meta-analysis was carried out to evaluate both the individual and synergistic effects of these factors. This article aims to investigate the interplay between MS and AP and explore their potential implications for clinical practice and public health.

## 2 Methods

We conducted our meta-analysis following the recommendations of the Cochrane Handbook (19) and the Preferred Reporting Items for Systematic Reviews and Meta-Analyses (PRISMA) 2020 guideline (20) (**Supplementary Table S1**). The protocol was registered on PROSPERO (21) (CRD42023471092), and we fully adhered to it. The research project is conducted under the Systems Education education-research model coordinated by the Centre for Translational Medicine at Semmelweis University (22).

### 2.1 Eligibility criteria

Eligibility criteria was determined according to the Population Exposure and Outcome (PEO) framework (23). We included studies reporting on adult patients with AP (P) and included MS and its four elements individually: obesity, hypertriglyceridemia (HTG), diabetes mellitus, and hypertension (E). Our outcomes (O) of interest were AP severity, in-hospital mortality, presence of local pancreatic complications (necrosis, fluid collection, pseudocyst), systemic complications (organ failure), and the need for Intensive Care Unit (ICU) admission. Articles in all languages and from all publication years were included to provide the most comprehensive overview possible. Studies reporting data on pediatric population, conference abstracts, case reports, case series, reviews, and meta-analyses were excluded. No other filters were used.

### 2.2 Search strategy

We systematically searched PubMed, Embase and the Cochrane Library on November 1, 2023. The search strategy combined terms related to *acute pancreatitis* and *metabolic syndrome* or its individual components (e.g., obesity, diabetes, hypertriglyceridemia, hypertension). The complete search strings for each database are provided in **Supplementary Methods S1**. No language or other restrictions were applied during the literature search. References of the included articles from the original pool investigating the effect of MS on AP outcome were also screened.

### 2.3 Selection process

After automatic and manual duplicate removal, the selection was performed first according to title and abstract using a specific selection tool (Rayyan) (24), then by full-text content by four independent reviewers (D.D. with A.C. and L.H. with D.C.). Cohen’s kappa coefficient was calculated after every selection step to assess the amount of disagreements that a third (B.Cs.B) independent reviewer subsequently solved.

### 2.4 Data collection process

The same four independent reviewers manually collected data and cross-checked it to ensure its quality using a standardized collection sheet. The extracted data included information about the study (first author, year of publication, country of origin, study design), demographic data (sample size, sex and age distribution), information on the exposure and the applied definition to determine its presence, and details about the outcomes (definition, frequency, time of measurement). We extracted the raw numbers, or the available odds ratios (ORs) and the corresponding 95% confidence intervals (CIs) as reported.

When analyzing the effect of overweight and obesity, we pooled all patients with a BMI over the normal range and created the group of patients with excess body weight (EBW).

We divided the included articles into two groups based on the applied cut-off value to determine HTG in AP patients. The first group contained articles where all elevated serum triglyceride levels above 150 mg/dL were included; we named it as “AP with HTG” group (8, 25–35). The second group consisted of studies where the exposed patients were diagnosed with “HTG-induced AP” (36–47) (triglyceride level at least 1000 mg/dL or 500 mg/dL with lactescent serum).

### 2.5 Risk of bias assessment

For the risk of bias assessment, we used the Quality in Prognostic Studies (QUIPS) tool (48). The evaluated domains included the number of study participants, study attrition, measurement of prognostic factors, outcome measurement, study confounding, and statistical analysis and reporting.

### 2.6 Synthesis methods

As we assumed considerable between-study heterogeneity in all cases, a random-effects model was used to pool effect sizes.

Odds ratio (OR) was used as the pooled effect size measure in our meta-analyses. To calculate the odds ratios and the pooled odds ratio, the total number of patients and those with the event of interest in each group (with and without MS, HTG, hypertension, diabetes or obesity) separately was extracted from the studies. We reported the results as the odds of event of interest in the “MS” group versus the odds of event of interest in the “no MS” group. We also reported the risks and risk differences for the individual studies for easier interpretation.

Some articles reported BMI, subcutaneous adipose tissue (SAT), and visceral adipose tissue (VAT) values across groups with different severities of pancreatitis. However, not all articles provided data for each severity group, and we aimed to compare all three severity categories. Therefore, as an additional form of analysis, we used a three-level (multilevel) meta-analysis model for these analyses.

Results were considered statistically significant if the pooled confidence interval (CI) did not contain the null value. We summarized the findings related to meta-analysis on forest plots. Between-study heterogeneity was described by the between-study variance (τ^2^) and the Higgins & Thompson’s I^2^ statistics too (49). We reported the prediction interval if at least eight studies were available for the meta-analysis.

The R software (50) was used for all statistical analyses with the meta (51) package for basic meta-analysis calculations and plots, and the dmetar (52) package for additional influential analysis calculations and plots. We reported additional, detailed information on calculations, data synthesis, publication bias assessment and influential analyses in **Supplementary Methods S2**. In this study, we examined the effects of MS and its components on AP outcomes through multiple analyses. Due to limitations on the number of figures and results that can be presented, we have included the most relevant findings in the main manuscript. Additional analyses and results, while not fully represented, are summarized and discussed in the **Supplementary Materials** for further reference.

## 3 Results

### 3.1 Search and selection

**Figure 1:**
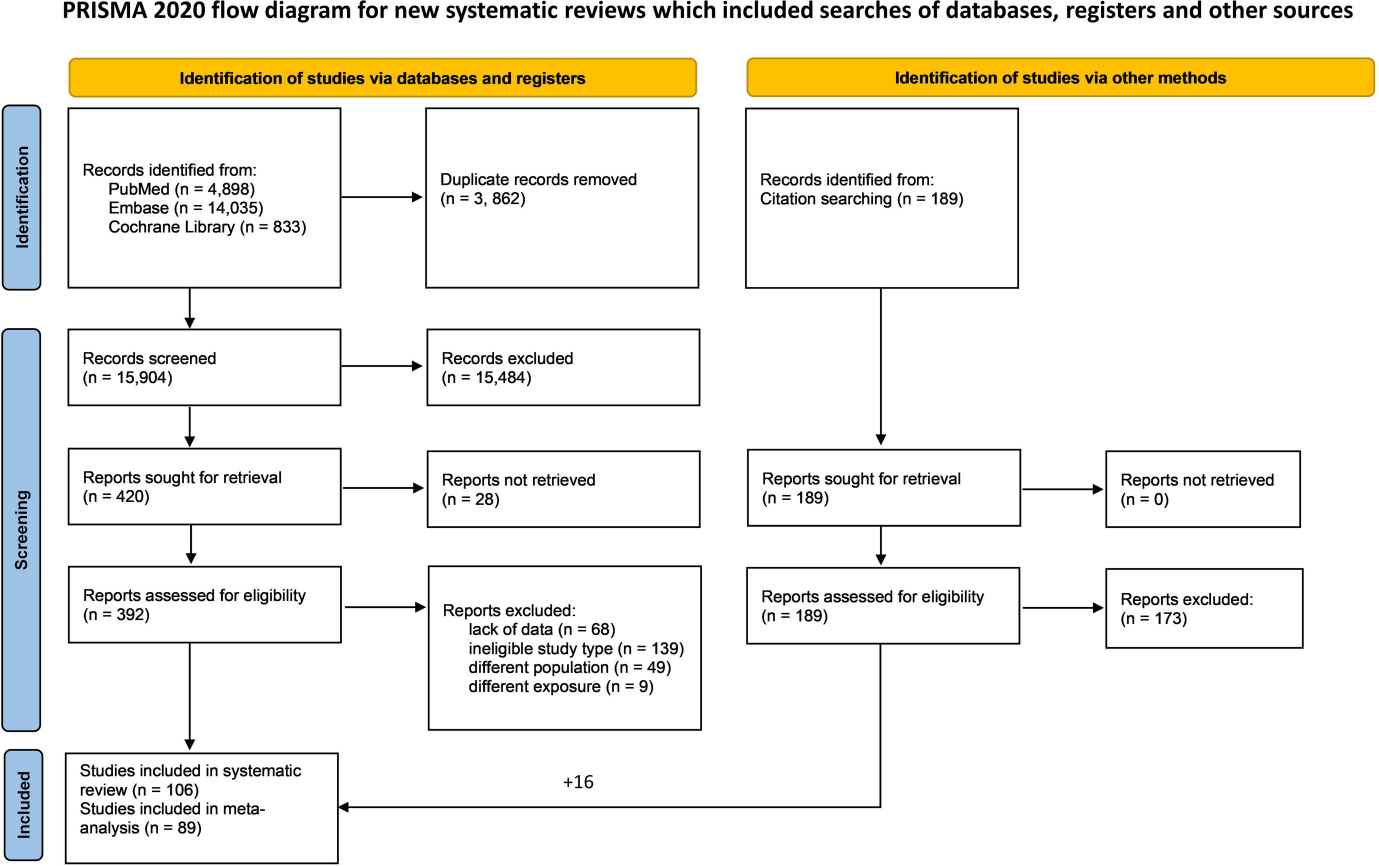
PRISMA flowchart of the selection process.

Our systematic search returned a total of 19,766 articles, and following automatic and manual duplicate removal, 15,904 records were screened. We assessed 392 full texts according to pre-defined eligibility criteria, the details of which are reported in **Supplementary Methods S3**. We included 106 articles in our systematic review, and 89 studies were eligible for meta-analysis (**Figure 1**).

### 3.2 Basic characteristics of the included studies

We included both prospective and retrospective observational studies. The sample sizes of the included articles ranged between 85 and 1,330,302. The definition of the investigated exposures was slightly different among the involved studies in some cases, so we performed subgroup analyses based on the applied method. In total, only 17 articles were included in the systematic review either because data reporting was ineligible to pool with the studies enrolled in the quantitative synthesis (27, 45, 53–62) or there was insufficient data on the specific exposure for statistical analysis (63–66). The basic characteristics of the enrolled studies are detailed in **Supplementary Table S2**.

### 3.3 Overweight and obesity

**Figure 2:**
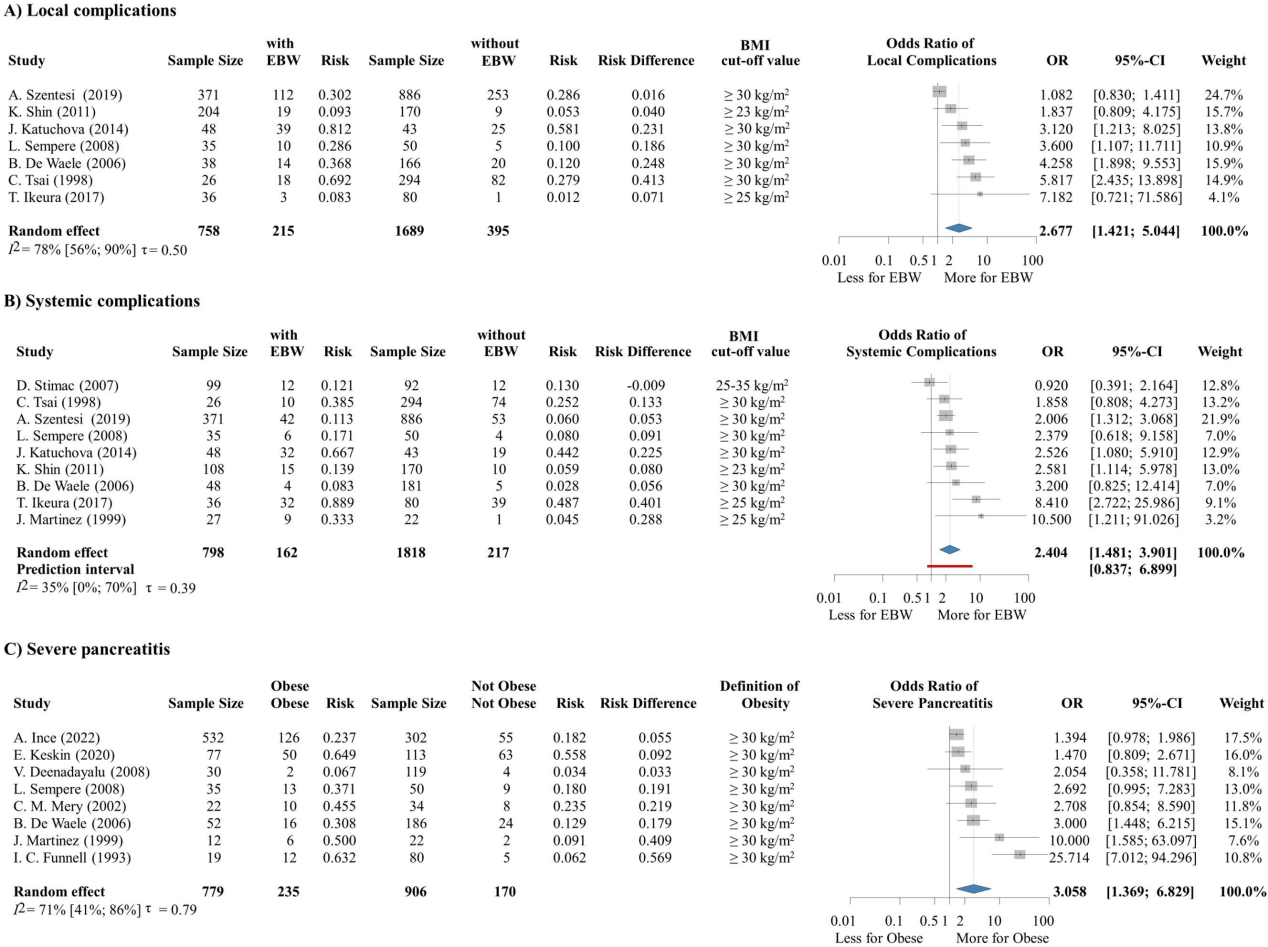
**(A)** The odds of local and **(B)** systemic complications is significantly increased in the group of patients with overweight or obesity. **(C)** The odds of developing severe AP is significantly increased among patients with obesity. EBW, excess body weight; BMI, body-mass index; OR, odds ratio; CI, confidence interval.

Seven studies (8, 67–72) including 1,814 patients reported data on the association between EBW and the development of local complications, while nine studies (8, 67–74) with a sample size of 2,616 reported on systemic complications. Overweight and obesity are found statistically significant predictors of local (OR: 2.677, CI: 1.421-5.044) (**Figure 2A**) and systemic (OR: 2.404, CI: 1.481-3.901) (**Figure 2B**) complications. Among these, renal failure (OR: 4.757, CI: 2.073-10.915) and respiratory failure (OR: 1.769, CI: 1.562-2.003) were significantly more frequent among patients with EBW (**Supplementary Figures S2**, **S3**). For disease severity and mortality, we performed a subset analysis using eligible articles that defined obesity according to the WHO’s 30 kg/m^2^ cut-off value. This subset involved 16 articles (8, 58, 67–71, 74–81) with an overall sample size of 4,449. We found that patients with obesity have a more than threefold increased risk of developing severe AP (OR: 3.058, CI: 1.369-6.829) (**Figure 2C**). The association between EBW and AP severity was also statistically significant when pooling together patients with moderately severe and severe AP – both outcomes were more common among patients with high BMI (OR: 2.276, CI: 1.353-3.828) (**Supplementary Figures S4**, **S5**).

We compared the mean BMI of patients in different severity groups with the mean values of two CT-based body composition parameters in the same group. We found that patients with moderately severe and severe AP have higher BMI and increased amount of VAT compared to patients with mild AP, however, we did not observe a difference in the SAT volumes of the groups (**Supplementary Figures S6**–**S9**).

### 3.4 Hypertension

**Figure 3:**
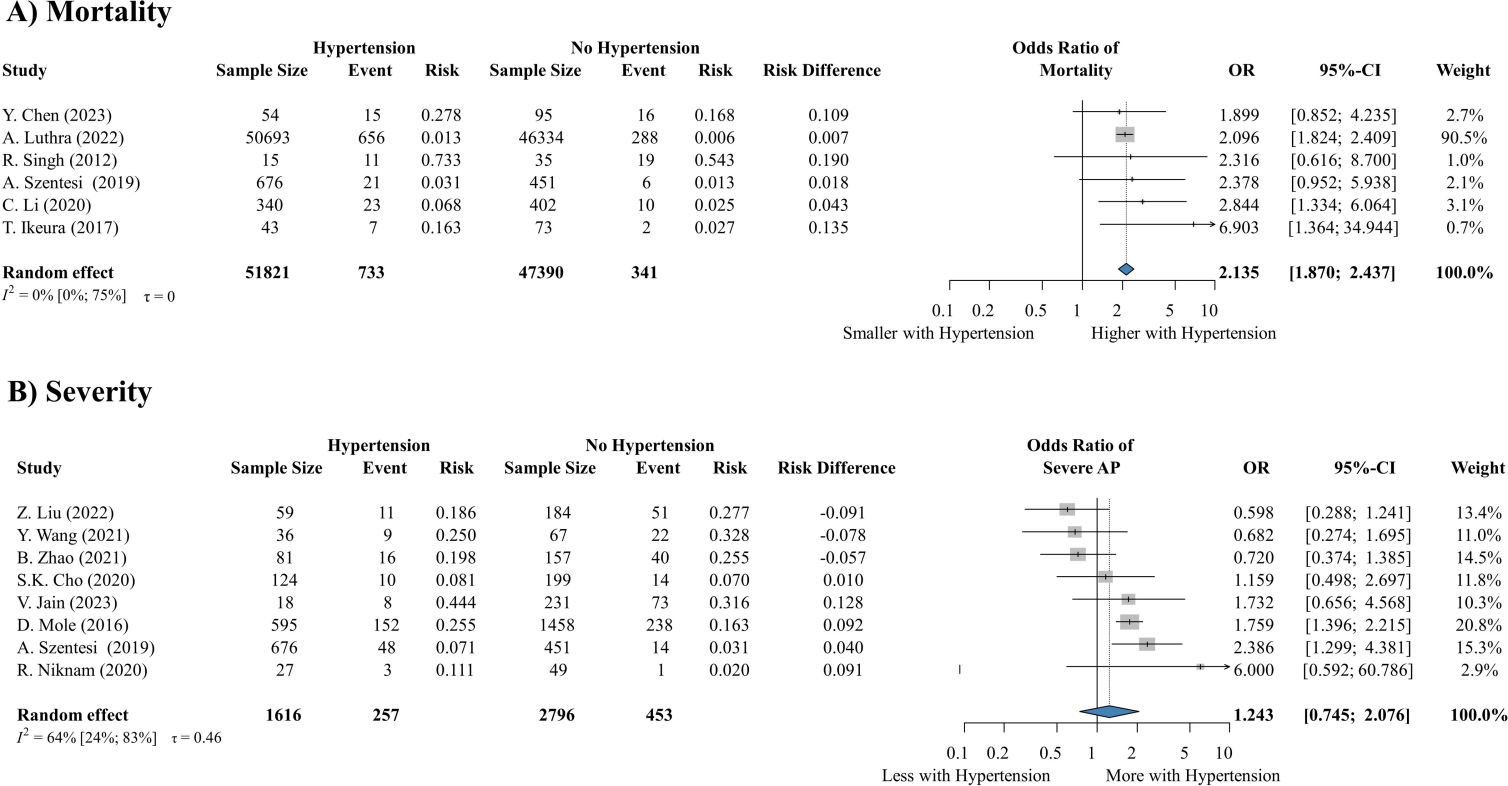
Hypertension significantly increases the risk of **(A)** mortality, but not severity **(B)**. AP, acute pancreatitis; OR, odds ratio; CI, confidence interval.

We were able to investigate the effect of pre-existing chronic hypertension on the outcome of AP based on data from 102,496 patients from 13 articles (8, 72, 82–92). According to our analysis, hypertension does not significantly increase the risk of severe AP (OR: 1.243, CI: 0.745-2.076), but it is a statistically significant predictor of mortality (OR: 2.135, CI: 1.870-2.437) (**Figure 3**).

### 3.5 Diabetes

**Figure 4:**
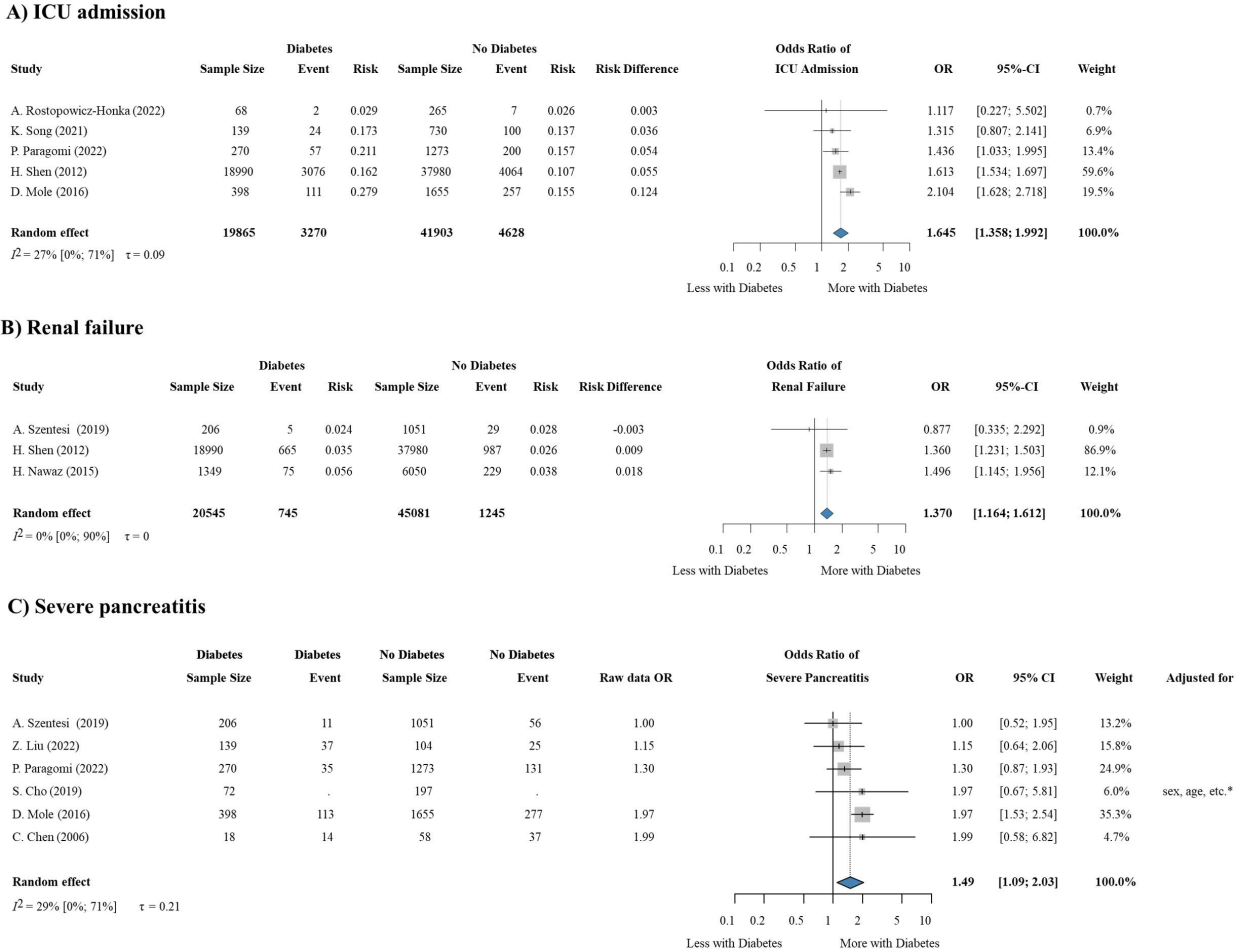
Diabetes is a statistically significant predictor of the need for **(A)** ICU admission, **(B)** renal failure and **(C)** severe pancreatitis. ICU, Intensive Care Unit; OR, odds ratio; CI, confidence interval.

In total, 11 articles (8, 27, 82, 87, 89, 92–97) containing data about 71,876 patients reported the outcomes of AP based on diabetes status. One of our major findings was that pre-existing diabetes mellitus is a risk factor of requiring ICU admission (OR: 1.645, CI: 1.358-1.992) (**Figure 4A**) and that of renal failure (OR: 1.370, CI: 1.164-1.612) (**Figure 4B**), and it remarkably increases the odds of severe AP as well (OR: 1.49, CI: 1.09-2.03) (**Figure 4C**). Diabetes also has a potential effect on mortality (OR: 2.373, CI: 0.320-17.570) (**Supplementary Figure S10**) and respiratory failure (OR: 1.159, CI: 0.653-2.058) (**Supplementary Figure S11**), however, these results were not statistically significant.

### 3.6 Hypertriglyceridemia

#### 3.6.1 AP with HTG

**Figure 5:**
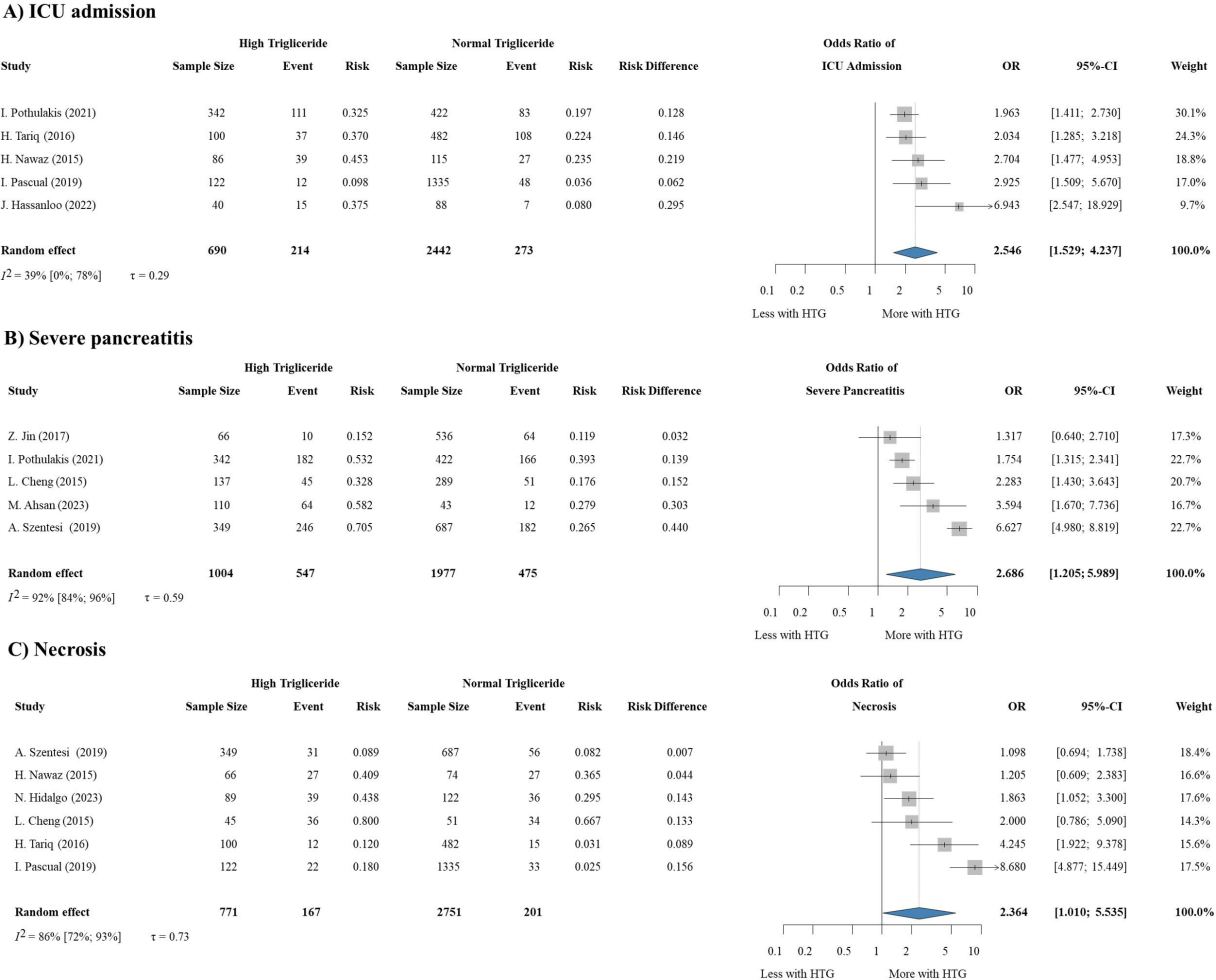
HTG is a predictor of **(A)** requiring ICU admission, **(B)** severity, and **(C)** necrosis development in AP. HTG, hypertriglyceridemia; OR, odds ratio; CI, confidence interval.

Based on 12 studies (8, 25–35) involving 291,733 patients, we could conclude that patients with elevated triglyceride levels on admission have significantly increased odds for being admitted to the ICU (OR: 2.546, CI: 1.529-4.237) (**Figure 5A**), progressing to the severe form of the disease (OR: 2.686, CI: 1.205-5.989) (**Figure 5B**) and developing necrosis (OR: 2.364, CI: 1.01-5.535) (**Figure 5C**).

#### 3.6.2 HTG induced AP

According to 13 studies (36–47) with an overall sample size of 581,787, patients with HTG-induced AP are more likely to have the severe form of pancreatitis (OR: 2.651, CI: 1.602-4.387) (**Supplementary Figure S12**) and develop necrosis during the course of the disease (OR: 1.709, CI: 1.070-2.728) (**Supplementary Figure S13**). HTG etiology has a potential effect on the odds of renal failure (OR: 2.780, CI: 0.531-14.547) and septic shock (OR: 1.91, CI: 0.65-5.62) as well, but these results were not statistically significant (**Supplementary Figures S14**, **S15**). The elevated risk of mortality in HTG-induced AP could not be proved in our analysis (OR: 0.987, CI: 0.490-1.988) (**Supplementary Figures S16**, **S17**).

### 3.7 Risk of bias assessment and sensitivity analyses

The risk of bias for attrition, prognostic factor measurement, outcome measurement, and study confounding was low in our analyses. Possible bias was observed in study participation and statistical analysis reporting. The results of our risk of bias assessment are available in **Supplementary Figure S1**.

Our leave-one-out analyses (where applicable) showed that no single study to be significantly influential, except for Funnel et al. (1993) (76) on the effect of obesity. Although Funnel et al. (1993) had some influence in some analyses, it did not materially change our conclusions.

### 3.8 Metabolic syndrome

**Figure 6:**
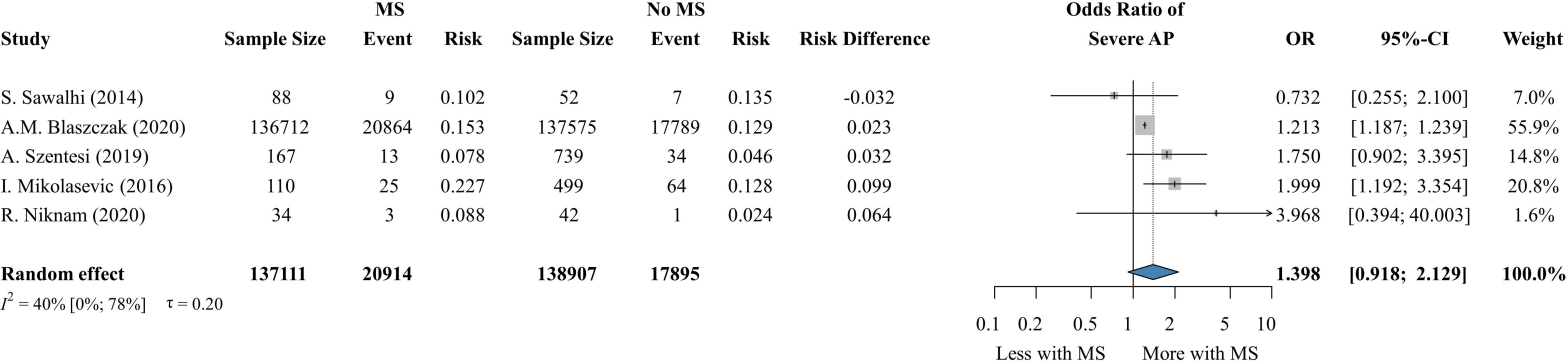
The risk of developing severe AP with and without MS. MS, metabolic syndrome; AP, acute pancreatitis; OR, odds ratio; CI, confidence interval.

Five articles (6, 8, 88, 98, 99) reported data on MS and AP severity (**Figure 6**), while 4 studies (6, 8, 88, 99) assessed the risk of mortality in the MS patient group. The overall sample size of the studies included was 137,111. Although the individual analyses of the components indicate an association between MS and AP severity, the odds of severe AP were not significantly increased in the patient group with MS (OR: 1.398, CI: 0.918-2.129) and we did not find a difference in the mortality of the exposed and non-exposed patient groups (**Supplementary Figure S18**).

## 4 Discussion

According to our analysis, all investigated individual components of MS are associated with a worse outcome in AP. In our earlier analysis, obesity was found to increase the risk of developing severe AP and even mortality (7), however our new findings highlight the connection between high BMI and the risk of both local and systemic complications.

Obesity is increasingly recognized as a significant risk factor for poorer outcomes in various health conditions due to its multifaceted impact on metabolic and inflammatory pathways. The enhanced accumulation of triglycerides and total fat in organs such as the liver and pancreas lead to insulin resistance, elevating the risk of necrosis and more severe abdominal pain, particularly in AP (100, 101). Moreover, the association between obesity and the development of diabetes is well-documented, implying a direct connection between excess body fat and metabolic dysregulation (102). Obesity is also characterized by low-grade chronic inflammation, marked by elevated levels of pro-inflammatory cytokines that can trigger systemic complications and worsen disease progression (103). Our findings support existing evidence, showing that obesity increases the risk of metabolic disorders and weakens immune responses, leading to worse clinical outcomes. Among the investigated factors, HTG was found to be a risk factor for disease severity, the need for ICU admission, and the development of necrosis, which is almost entirely in accordance with earlier analyses (11, 104). Previous studies have linked HTG to a higher risk of mortality as well. However, in our analysis, eight of the included studies found a connection between HTG and mortality in AP, while three studies (including one large retrospective cohort (30)) did not. As a result, we were unable to confirm the previous findings on this issue in our meta-analysis (**Supplementary Figure S13**). We were planning to analyze the prognostic role of other lipid parameters, such as high and low-density lipoproteins (HDL and LDL) as well. We did not have enough data from the included articles to perform a statistical analysis focusing on this question, but a cohort study associated abnormal cholesterol concentrations with longer hospital stay, and an elevated risk of mortality, too (66).

Preexisting diabetes was known to lead to a poor outcome of AP, and our results confirm the findings of previous studies about its role in elevating the risk of renal failure and the need for ICU admission (13).

In an earlier cohort study, hypertension was associated with an increased risk of severe AP and systemic complications (8), while we observed an elevated chance for a fatal outcome. Hypertension may exacerbate the outcome of AP through vascular and microcirculatory dysfunctions associated with chronic high blood pressure (105). Furthermore, recent studies have demonstrated that persistent hypertension elevates oxidative stress in the pancreas. This oxidative stress is a key factor in pancreatic inflammation and is ultimately linked to the onset of pancreatitis as well (106, 107).

Although the deteriorating effect of the individual MS components was clear in our analysis we did not find a significant association between MS and AP outcome. This discrepancy may be explained by the heterogeneous definitions of MS across studies, the unweighted combination of its components that may dilute the strong effect of certain factors, and the relatively small number of studies directly investigating MS as a whole. These factors together may have reduced the statistical power to detect a significant association.

We have focused on the in-hospital outcome of AP – due to the limited data on its long-term consequences – but evaluating the post-discharge outcomes would also be crucial. In our recent analysis, we showed that pre-existing cardiovascular diseases and a more severe course of AP can be associated with post-discharge mortality (108). Therefore, to prevent or minimize the late consequences of AP, it would be important to follow up patients, especially those with obesity and any associated comorbidities having a higher risk of a more severe disease course.

### 4.1 Strengths and limitations

As for the strengths of our analysis, we followed our protocol, which was registered in advance, and applied a rigorous methodology. We performed a comprehensive analysis of a multifactorial condition and assessed the effect of its individual components as well. This study is the first meta-analysis to assess the effect of hypertension on the outcomes of AP, highlighting a significant strength of our research. We showed that obesity worsens both local and systemic complications.

Among the limitations, we should note that we included both prospective and retrospective observational studies, and national databases that often lack a uniform protocol for data collection, which can result in lower data quality. Since the study number was limited, the publication bias and the influence assessment of individual studies have limited diagnostic value. Moreover, this small sample size makes the estimation of heterogeneity highly uncertain. Heterogeneity may also stem from variability in how MS itself was defined across the included studies. Because different diagnostic criteria were applied in different settings (15), direct comparison between studies was challenging. Differences in study design (e.g., prospective *vs*. retrospective), study populations (including age distribution, comorbidity burden, and severity spectrum), and regional baseline risks (such as prevalence of metabolic comorbidities and healthcare system characteristics) may further contribute to the observed variability. Due to these inconsistencies and the way data were reported, we were unable to stratify our analyses according to specific definitions. Instead, all studies were analyzed together, which we acknowledge as a methodological limitation. Future research using standardized definitions and harmonized reporting of metabolic parameters could allow a more nuanced evaluation of their impact on AP outcomes. Although BMI is currently the most commonly used tool for assessing obesity, it lacks accuracy as it does not account for muscle mass, fat distribution, or variations in body composition, leading to potential misclassifications.

### 4.2 Implication for research and practice

Our research delivers important insights that have the potential to shape daily clinical practices. Consequently, it qualifies as translational medicine, impacting both scientific inquiry and practical applications (109, 110).

Across the major prognostic models for AP, none systematically incorporate the core components of MS. Although the Ranson (111) and Glasgow–Imrie criteria (112) include admission glucose as a marker of dysglycemia, most of the scores (113–118) omit obesity, triglycerides or chronic hypertension. Only the recently developed EASY-APP (18) integrates glucose and allows entry of BMI or blood pressure into its model, but triglycerides remain absent, and metabolic features are not among its strongest predictors. Our findings indicate that obesity and hypertriglyceridemia are among the most powerful determinants of adverse outcomes, underscoring a gap across all currently applied systems. Future research should therefore examine whether incorporating these metabolic parameters into established or next-generation scores could enhance predictive accuracy and enable earlier, more targeted decision-making.

Patients with MS or its components have a high risk of a poor AP outcome, and therefore require closer monitoring. Considering the high rates of AP recurrence, lifestyle counseling and education of AP patients with obesity is crucial. However, better treatment strategies for overweight and obesity are needed as well to prevent the formation of concomitant metabolic abnormalities; even primary care should address obesity directly, not only the related comorbidities.

Despite the strong link between MS and AP, gaps in the literature persist, particularly regarding causal mechanisms. Future research should prioritize explaining the mechanisms connecting these two conditions to improve clinical practices and public health initiatives.

## 5 Conclusion

Components of metabolic syndrome are risk factors for complications, severity, and mortality in acute pancreatitis.

## Data Availability

Publicly available datasets were analyzed in this study. This data can be found here: The datasets used in this study can be found in the full-text articles included in the systematic review and meta-analysis.
