# Supplementary material for: Metabolic syndrome components individually worsen the outcome of acute pancreatitis: a systematic review and meta-analysis
Source: Front Endocrinol (Lausanne). 2025 Nov 4;16:1690754. doi: 10.3389/fendo.2025.1690754 (PMC12623212; doi:10.3389/fendo.2025.1690754)
Supplement: Supplementary file 1 [file Supplementaryfile1.pdf]

Supplementary Material

Metabolic Syndrome Components Individually Worsen The Outcome of Acute Pancreatitis: A Systematic Review and Meta-analysis

Dalma Dobszai[1,2], Mahmoud Obeidat[2], Eszter Ágnes Szalai[2,3], Dániel Sándor Veres[2,4], Luca Havelda[2,5], Renáta Papp[2,6,7], Alim Choi[2], Bettina Csilla Budai[2,5], Dominika Csajbók[2], Péter Hegyi[1,2,5,8*], Andrea Szentesi[1,2*]

*Contributed equally.

1. Institute for Translational Medicine, Medical School, University of Pécs, Pécs, Hungary
2. Centre for Translational Medicine, Semmelweis University, Budapest, Hungary
3. Department of Restorative Dentistry and Endodontics, Semmelweis University, Budapest, Hungary
4. Department of Biophysics and Radiation Biology, Semmelweis University, Budapest, Hungary
5. Institute of Pancreatic Diseases, Semmelweis University, Budapest, Hungary
6. Department of Pharmacology and Pharmacotherapy, Semmelweis University, Budapest, Hungary
7. Center for Pharmacology and Drug Research & Development, Semmelweis University, Budapest, Hungary
8. Translational Pancreatology Research Group, Interdisciplinary Centre of Excellence for Research Development and Innovation, University of Szeged, Szeged, Hungary

Document Legends

Supplementary Table 1.: PRISMA 2020 Checklist ………………………………………………………………………………………… 3
Supplementary Methods 1.: Individualised search key in different databases ……..……………………………………………..………….. 6
Supplementary Methods 2.: Additional details on data synthesis ………………………………………………………………………... 7
Supplementary Methods 3.: Detailed strategy for selection and retrieval ………………………………………………………………... 8
Supplementary Table 2.: Basic characteristics of the included studies ……………………………………………………….……….. 9
Supplementary Figure 1.: Risk of bias assessment ………………………………………………………………………………………… 16
Supplementary Figure 2.: The odds of developing renal failure with and without excess body weight (EBW) …………………………………... 18
Supplementary Figure 3.: The odds of developing respiratory failure with and without EBW ……………………………………………………… 18
Supplementary Figure 4.: The odds of developing moderately severe or severe pancreatitis with and without EBW ………………………….. 19
Supplementary Figure 5.: Funnel plot corresponding to the odds of developing moderately severe or severe pancreatitis with and without EBW …. 20
Supplementary Figure 6.: The mean body-mass index (BMI) in the different AP severity groups ………………………………………… 21
Supplementary Figure 7.: Funnel plot corresponding to the mean body-mass index (BMI) in the different AP severity groups …………….…. 22
Supplementary Figure 8.: The mean volume of subcutaneous adipose tissue (SAT) in the different AP severity groups …………….…………. 23
Supplementary Figure 9.: The mean volume of visceral adipose tissue (VAT) in the different AP severity groups ………………………….. 24
Supplementary Figure 10.: The odds of mortality with and without diabetes ………………………………………………………………... 25
Supplementary Figure 11.: The odds of respiratory failure with and without diabetes ……………………………………………………….. 25
Supplementary Figure 12.: The odds of developing severe AP with and without HTG etiology ……………………………………………………. 26
Supplementary Figure 13.: The odds of developing necrosis with and without HTG etiology ……………………………………………………. 27
Supplementary Figure 14.: The odds of developing renal failure with and without HTG etiology ……………………………………………………. 27
Supplementary Figure 15.: The odds of falling into septic shock with and without HTG etiology ……………………………………………………. 28
Supplementary Figure 16.: The odds of mortality in AP with and without HTG etiology ……………………………………………………….. 29
Supplementary Figure 17.: Funnel plot corresponding to the odds of mortality in AP with and without HTG etiology ………………...……… 30
Supplementary Figure 18.: The odds of mortality in the groups of patients with and without metabolic syndrome (MS) ………………...……… 31

Supplementary Table 1.: PRISMA 2020 Checklist

Section and Topic	Item #	Checklist item	Item is reported
**TITLE**			
Title	1	Identify the report as a systematic review.	✓
**ABSTRACT**			
Abstract	2	See the PRISMA 2020 for Abstracts checklist.	✓
**INTRODUCTION**			
Rationale	3	Describe the rationale for the review in the context of existing knowledge.	✓
Objectives	4	Provide an explicit statement of the objective(s) or question(s) the review addresses.	✓
**METHODS**			
Eligibility criteria	5	Specify the inclusion and exclusion criteria for the review and how studies were grouped for the syntheses.	✓
Information sources	6	Specify all databases, registers, websites, organisations, reference lists and other sources searched or consulted to identify studies. Specify the date when each source was last searched or consulted.	✓
Search strategy	7	Present the full search strategies for all databases, registers and websites, including any filters and limits used.	Supplementary Methods 3
Selection process	8	Specify the methods used to decide whether a study met the inclusion criteria of the review, including how many reviewers screened each record and each report retrieved, whether they worked independently, and if applicable, details of automation tools used in the process.	✓
Data collection process	9	Specify the methods used to collect data from reports, including how many reviewers collected data from each report, whether they worked independently, any processes for obtaining or confirming data from study investigators, and if applicable, details of automation tools used in the process.	✓
Data items	10a	List and define all outcomes for which data were sought. Specify whether all results that were compatible with each outcome domain in each study were sought (e.g. for all measures, time points, analyses), and if not, the methods used to decide which results to collect.	✓
	10b	List and define all other variables for which data were sought (e.g. participant and intervention characteristics, funding sources). Describe any assumptions made about any missing or unclear information.	✓
Study risk of bias assessment	11	**Specify the methods used to assess risk of bias in the included studies, including details of the tool(s) used, how many reviewers assessed each study and whether they worked independently, and if applicable, details of automation tools**	✓

Section and Topic	Item #	Checklist item	Item is reported
		**used in the process.**	
Effect measures	12	Specify for each outcome the effect measure(s) (e.g. risk ratio, mean difference) used in the synthesis or presentation of results.	✓
Synthesis methods	13a	Describe the processes used to decide which studies were eligible for each synthesis (e.g. tabulating the study intervention characteristics and comparing against the planned groups for each synthesis (item #5)).	✓
	13b	Describe any methods required to prepare the data for presentation or synthesis, such as handling of missing summary statistics, or data conversions.	✓
	13c	Describe any methods used to tabulate or visually display results of individual studies and syntheses.	✓
	13d	Describe any methods used to synthesize results and provide a rationale for the choice(s). If meta-analysis was performed, describe the model(s), method(s) to identify the presence and extent of statistical heterogeneity, and software package(s) used.	✓
	13e	Describe any methods used to explore possible causes of heterogeneity among study results (e.g. subgroup analysis, meta-regression).	✓
	13f	Describe any sensitivity analyses conducted to assess robustness of the synthesized results.	✓
Reporting bias assessment	14	Describe any methods used to assess risk of bias due to missing results in a synthesis (arising from reporting biases).	✓
Certainty assessment	15	Describe any methods used to assess certainty (or confidence) in the body of evidence for an outcome.	N/A
**RESULTS**			
Study selection	16a	Describe the results of the search and selection process, from the number of records identified in the search to the number of studies included in the review, ideally using a flow diagram.	✓
	16b	Cite studies that might appear to meet the inclusion criteria, but which were excluded, and explain why they were excluded.	✓
Study characteristics	17	Cite each included study and present its characteristics.	Supplementary Table 2
Risk of bias in studies	18	Present assessments of risk of bias for each included study.	Supplementary Figure 1
Results of individual studies	19	For all outcomes, present, for each study: (a) summary statistics for each group (where appropriate) and (b) an effect estimate and its precision (e.g. confidence/credible interval), ideally using structured tables or plots.	✓
Results of syntheses	20a	For each synthesis, briefly summarise the characteristics and risk of bias among contributing studies.	✓
	20b	Present results of all statistical syntheses conducted. If meta-analysis was done, present for each the summary estimate and its precision (e.g. confidence/credible interval) and measures of statistical heterogeneity. If comparing groups, describe the direction	✓

Section and Topic	Item #	Checklist item	Item is reported
		of the effect.	
	20c	Present results of all investigations of possible causes of heterogeneity among study results.	✓
	20d	Present results of all sensitivity analyses conducted to assess the robustness of the synthesized results.	N/A
Reporting biases	21	Present assessments of risk of bias due to missing results (arising from reporting biases) for each synthesis assessed.	Supplementary Figure 1
Certainty of evidence	22	Present assessments of certainty (or confidence) in the body of evidence for each outcome assessed.	N/A
**DISCUSSION**			
Discussion	23a	Provide a general interpretation of the results in the context of other evidence.	✓
	23b	Discuss any limitations of the evidence included in the review.	✓
	23c	Discuss any limitations of the review processes used.	✓
	23d	Discuss implications of the results for practice, policy, and future research.	✓
**OTHER INFORMATION**			
Registration and protocol	24a	Provide registration information for the review, including register name and registration number, or state that the review was not registered.	✓
	24b	Indicate where the review protocol can be accessed, or state that a protocol was not prepared.	✓
	24c	Describe and explain any amendments to information provided at registration or in the protocol.	✓
Support	25	Describe sources of financial or non-financial support for the review, and the role of the funders or sponsors in the review.	✓
Competing interests	26	Declare any competing interests of review authors.	✓
Availability of data, code and other materials	27	Report which of the following are publicly available and where they can be found: template data collection forms; data extracted from included studies; data used for all analyses; analytic code; any other materials used in the review.	✓

From: Page MJ, McKenzie JE, Bossuyt PM, Boutron I, Hoffmann TC, Mulrow CD, et al. The PRISMA 2020 statement: an updated guideline for reporting systematic reviews. BMJ 2021;372:n71. doi: 10.1136/bmj.n71

Supplementary Methods 1.: Individualised search key in different databases

PubMed:

(("acute" OR "biliary") AND "pancreatitis") AND (metabolic syndrome OR "MetS" OR "hypertension" OR "diabet*" OR "diabetes" OR insulin resistance OR fasting glucose OR waist circumference OR "WC" OR "HDL" OR "cholesterol" OR "obes*" OR "obesity" OR "overweight" OR "hypertriglyceridemia")

EMBASE:

((acute OR biliary) AND pancreatitis) AND ((metabolic syndrome) OR MetS OR hypertension OR diabet* OR diabetes OR (insulin resistance) OR (fasting glucose) OR (waist circumference) OR WC OR HDL OR cholesterol OR obes* OR obesity OR overweight OR hypertriglyceridemia)

Cochrane Library:

((acute OR biliary) AND pancreatitis) AND (metabolic syndrome OR MetS OR hypertension OR diabet* OR diabetes OR insulin resistance OR fasting glucose OR waist circumference OR WC OR HDL OR cholesterol OR obes* OR obesity OR overweight OR hypertriglyceridemia)

Supplementary Methods 2.: Additional details on data synthesis

For pooling the effect size in “2 level models”, pooled OR was calculated by the Mantel-Haenszel method [1, 2]. Exact Mantel-Haenszel method (without continuity correction) was used to handle zero cell counts (as recommended by Cooper and Sweeting [3, 4]). We used a Hartung-Knapp adjustment [5, 6] for confidence intervals (CIs). This adjustment was applied only if it is more conservative then the classical one (as recommended in Jackson et al. [7] as hybrid method 2). To estimate the heterogeneity variance measure (τ^2), the Paule-Mandel method [8] was used with the Q profile method for confidence interval (recommended in Harrer et al. and Veroniki et al. [9, 10]).

In the three-level analyses, we accounted for estimates derived from the same article (but classified into different categories) by including an additional random-effects term, thereby considering their correlation. For the pooled estimates of means in these models, we used the inverse variance method, with random effects estimated using the restricted maximum likelihood approach. Confidence intervals were calculated using the t-distribution. For severity group comparisons, we provided Wald-type p-values, and for pairwise comparisons between groups, we reported p-values adjusted for multiplicity using the Holm method. In case of the multilevel model, we reported the I^2 statistics for each level with its 95% CI as given in Cheung et al.[11]. We refer as "total I^2" for the sum of the two level as the total heterogeneity over sampling variance.

Small study publication bias was assessed by visual inspection of Funnel-plots and calculating (for the binary outcomes) Harbord test p-value [12]. We planned to assume possible small study bias if the p-value is less than 10%. (Although, we kept in mind that the test has limited diagnostic assessment below ~10 study.)

Potential outlier publications was explored using different influence measures - eg. we calculated dfbetas (the pooled effect size without the given study - expressed in logit scale for binary data), Cook's distances (shows how much the estimated effect size changes leaving-out the given study, but taking into consideration of how much differ the leaved-out study effect size from the pooled effect size. - typical threshold for potential influential value is 2) and hat values (the value of the hat matrix without the given study.) leaving out 1 study at a time - and plotting them following the recommandation of Harrer et. al [9] using the dmetar package. In the case of multilevel analyses, we calculated dfbetas, Cook's distances and hat values, leaving out 1 study at a time.

Supplementary Methods 3.: Detailed strategy for selection and retrieval

We have emailed the corresponding authors.
During the full text selection, we excluded 304 articles because of the following reasons: 70 - no data regarding exposures were reported, 49 ineligible population, 37 – unreported outcome of interest, 139 - ineligible study type and 9 – different exposure was investigated.
During the manual data retrieval, if odds ratio (OR) with the corresponding 95% confidence interval (Cl) were not reported, the available raw data were used (which is the number of patients in each group of interest-exposed patients experiencing the outcome, exposed patients not experiencing the outcome, non-exposed patients experiencing the outcomes, and non-exposed patients not experiencing the outcome, respectively).

Supplementary Table 2.: Basic characteristics of the included studies

Study	Year of publication	Study site	Number of patients (female %)	Age (year) ‡	Analyzed prognostic factor	Measurement tool/Definition
Ahsan et al.	2023	Banglades h	153 (48%)	46.72±13.43	HTG	>150 mg/dL
Baranyai et al.	2012	Hungary	351 (46%)	HTG group: 40.5±1.8 non-HTG group:60±0.9	HTG	>11.3 mmol/l
Beydogan et al.	2021	Turkey	174 (61%)	58.7±18.3	obesity	abdominal fat distribution parameters measured with computed tomography (CT)
Biberci-Keskin et al.	2020	Turkey	190 (62%)	73 (68-79)	obesity	BMI≥30
Blaszczak et al.	2020	USA	1078183 (NA)	N/A	obesity, MetS	BMI≥40, presence of at least 3 factors
Bosques-Padilla et al.	2015	Mexico	191 (62%)	39.06±15.8	HTG	1000 mg/dL
Bota et al.	2013	Romania	529 (40%)	54.3±16.4	obesity	BMI
C. Chen et al.	2006	Taiwan	106 (20%)	43.5±12.9	HTG	>500 mg/dL
Chaigneau et al.	2023	France	467 (34%)	57±19	obesity	BMI
Cheng et al.	2015	China	426 (56%)	HTG group: 46.35±7.26 non-HTG group: 54.89±7.84	HTG	≥1.88 mmol/L
Cho et al.	2020	South Korea	323 (38%)	56.2±18.3	HTG	N/A
Dancu et al.	2022	Romania	262 (43%)	57±17	HTG	1000 or 500mg/dL

Davis et al.	2012	Canada	45 (20%)	obese group: 58.5±13.9 nonobese group: 60.4±12.8	obesity	BMI≥30
De Waele et al.	2006	Belgium	238 (62%)	obese group: 63.5±1.3 nonobese group: 62.8±2.0	obesity	BMI≥30
Deenadayalu et al.	2008	USA	964 (64%)	obese group: 48.8±15.2 nonobese group: 51.6±16.9	obesity	BMI≥30
Deng et al.	2008	China	176 (44%)	HTG group: 40.8±9.3 control group: 52.6±13.4	HTG	TG ≥ 5.65 mmol/L
Duarte-Rojo et al.	2010	Mexico	99 (51%)	44±16	obesity	BMI, abdominal fat distribution parameters measured with computed tomography (CT)
Funnell et al.	1993	South Africa	99 (31%)	41.2±12.7	obesity	BMI≥30
Goyal et al.	2016	USA	177 (34%)	49.47±10.89	HTG	>1000 mg/dL
Hassan et al.	2021	India	80 (28%)	18-68	obesity	BMI
Hassanloo et al.	2022	Canada	128 (41%)	HTG group: 51±14 control group: 50±14	HTG	≥ 5.6 mmol/L
Hidalgo et al.	2023	Spain	211 (38%)	no necrosis: 64.53±19.13 necrosis: 63.48±16.24	HTG	Normal TG (< 150 mg/dL), Borderline-high TG (150–199 mg/dL), High TG (200–499 mg/dL), Very high TG group (≥ 500 mg/dL)
Hong et al.	2017	China	647 (37%)	47 (37-63)	HTG	>1000 mg/dL
Huang et al.	2014	China	1582 (34%)	hyperlipidemic AP: 40.8±10.9 biliary AP: 58.8±15.6	HTG	>11.3 mmol/L or above 5.6 mmol/L with lactescent serum on admission
Ikeura et al.	2017	Japan	116 (33%)	61.6±15.1	obesity	BMI≥25
Ince et al.	2022	Turkey	1334 (54%)	54.96±17.48	obesity	BMI≥30

Jain et al.	2023	India	249 (30%)	39±14.4 to 43.4±16.02	diabetes, hypertension	NA
Jang et al.	2021	South Korea	242 (11%)	47.0±12.6	obesity	BMI, abdominal fat distribution parameters measured with computed tomography (CT)
Jiang et al.	2005	China	99 (40%)	HTG: 48.3 ± 14.9 normal: 60.9 ± 14.9	HTG	serum TG >1.7 mmol/L
Jin et al.	2017	China	602 (57%)	54±17	obesity	BMI≥25
Karpavicius et al.	2016	Lithuania	102 (51%)	55.7±18.1	obesity	BMI
Katuchova et al.	2014	Slovakia	91 (38%)	obese group: 53.47±3.88 nonobese group: 54.32±4.09	obesity	BMI≥30
Kim et al.	2020	South Korea	499 (55%)	HTG group: 36.92±10.12 non-HTG group: 51.62±17.41	HTG	> 1000 mg/dL
Krishna et al.	2015	USA	1330302 (48%)	obese group: 48.64±15.02 nonobese group: 52.47±17.44	obesity	BMI>40
L. Chen et al.	2021	China	242 (20%)	40 (34-47)	obesity	BMI, abdominal fat distribution parameters measured with computed tomography (CT)
Lee et al.	2023	USA	1544 (48%)	49.63±18.47	obesity	BMI≥30
Li et al.	2018	China	730 (38%)	HTG AP: 40 (33-47) non-HTG AP: 51 (43-64)	HTG	> 1000 mg/dL
Li et al.	2020	China	742 (39%)	Survived: 51.00 (40.00-63.00) Died: 65.00 (60.00-78.00)	HT, diabetes	NA

Liu et al.	2022	China	243 (23%)	40.12±10.12	HT, BMI, diabetes	NA
Luo et al.	2023	China	496 (26%)	Modeling group: 47.4 (45.6, 49.3) Validation group: 46.1 (43.7, 48.5)	HT, diabetes	NA
Luthra et al.	2022	USA	97027 (62%)	mortality:75.2±13.1 no mortality: 57.2±19.6	HT, diabetes	NA
Martinez et al.	1999	Spain	49 (49%)	59.1±18.8	obesity	BMI≥30
McGuire et al.	2022	USA	536 (35%)	obese group: 51.6±15.4 nonobese group: 52.2±15.6	obesity	BMI>30
Mery et al.	2002	Mexico	88 (60%)	43.6±17.9	obesity	BMI>30
Mery et al.	2002	Mexico	150 (51%)	N/A	obesity	BMI≥25
Mikolasevic et al.	2016	Croatia	609 (44%)	63.2±16.1	MetS	International Diabetes Federation criteria
Mole et al.	2016	Scotland	2053 (50%)	55.8±18.5	HT, diabetes	ICD-10 codes
Moran et al.	2018	Spain	1655 (46%)	64.5±17.3	obesity	BMI≥30
Navarro et al.	2004	Spain	38 (55%)	HTG AP: 42.5±11.4 non-HTG AP:43.3±3.9	HTG	>1000 mg/dL
Nawaz et al.	2015	USA	7399 (56%)	57 (43-74)	diabetes	ICD-9 codes
Niknam et al.	2020	Iran	76 (47%)	47.9±15.7	MetS	NCEP/ATP-III criteria
O'Leary et al.	2012	Ireland	62 (38%)	51.3±16.7	obesity	abdominal fat distribution parameters measured with computed tomography (CT)
Papachristou et al.	2005	USA	102 (51%)	50 (15-90)	obesity	BMI≥30
Paragomi et al.	2022	USA	1543 (48%)	49.6±18.5	diabetes	NA

Pascual et al.	2019	Spain	1457 (51%)	Normal TG: 68.0 (17-98) Moderate HTG: 49.9 (30-92) Severe HTG: 41.9 (28-72)	HTG	Moderate HTG (TG 200-749mg/dl) Severe HTG (TG≥750mg/dl)
Podda et al.	2022	Italy	5275 (52%)	63 ± 19	HT, diabetes	NA
Pothoulakis et al.	2021	USA	764 (42%)	NA	HTG	TG≥200mg/dl
Rostopowicz-Honka et al.	2022	Poland	333 (42%)	NA	diabetes	
Sawalhi et al.	2014	Saudi Arabia	140 (57%)	48.4	obesity	BMI
Sempere et al.	2008	Spain	85	63.7±17.4	obesity	BMI≥30
Sezgin et al.	2019	Turkey	635 (54%)	HTG AP: 37.6±14.8 non-HTG AP: 57.4±17.3	HTG	N/A
Shafiq et al.	2022	India	550 (22%)	HTG AP: 34.3±5.12 non-HTG AP: 46.27±19.68	HTG	>1000 mg/dL
Shaka et al.	2020	USA	575230 (NA)	HTG-AP: 56.7, Non-HTG AP: 50.8	HTG	Secondary diagnosis of HTG based on ICD-10 codes
Sharma et al.	2009	USA	128 (54%)	51.1±19.8	obesity	BMI
Shen et al.	2012	Taiwan	1131927 (52%)	NA	diabetes	
Shin et al.	2011	South Korea	374 (31%)	mild AP group: 55.8±0.9 severe AP group: 54.8±1.6	obesity	BMI≥30
Simons-Linares et al.	2019	USA				

Song et al.	2021	China	872 (37%)	HTG AP: 37 (32-45) non-HTG AP: 51 (41-65)	HTG	≥11.3 mmol/L or 5.65 mmol/L accompanied by milky serum
Song et al.	2023	China	872 (37%)	Non-HTG AP: 51 (41-65) HTG AP: 37 (32-45)	HTG	serum TG was ≥11.3 mmol/L or ≥5.65 mmol/L accompanied by milky serum
Stimac et al.	2007	Croatia	357 (64%)	60.4±15.0	obesity	BMI≥30
Szentesi et al.	2019	Hungary	1127 (43%)	55.7±17	obesity, MetS	BMI≥30, presence of at least 3 factors
Taguchi et al.	2014	Japan	6002 (35%)	64±17.5	obesity	BMI≥30
Tariq et al.	2016	USA	582 (39%)	HTG:50.5±13 Non-HTG: 49±14	HTG	triglyceride level of ≥2.26mmol/L
Thandassery et al.	2014	India	280 (29%)	40.67±13.12	obesity	BMI≥25
Thong et al.	2021	Vietnam	157 (22%)	41.5±9.7	HTG	triglyceride level of ≥1000mg/dL
Tsai et al.	1998	Taiwan	320 (37%)	51.1±16.6	obesity	BMI≥30
Türkoglu et al.	2014	Turkey	92 (76%)	52.5±19.6	obesity	BMI
Wang et al.	2021	China	103 (42%)	46 (34-60)	hypertension	NA
Wu et al.	2014	China	245 (40%)	AKI group: 46.4±12.4 non-AKI group:46.3±15.1	HTG	≥11.3 mmol/L or more than 5.65 mmol/L with a lactescent serum
Wu et al.	2018	China	99 (34%)	48.71±13.97	HTG	TG levels greater than 11.3 mmol/L or TG levels greater than 5.65 mmol/L with grossly lipaemic serum
Xie et al.	2019	China	306 (49%)	50.6±18.7	obesity	BMI, abdominal fat distribution parameters measured with computed tomography (CT)
Y. Chen et al.	2023	China	149 (38%)	38.30 ± 12.63	hypertension, diabetes	NA
Yang et al.	2017	China	96 (45%)	41	obesity	BMI≥28

Yashima et al.	2011	Japan	124 (40%)	59.7±15.6	obesity	BMI, abdominal fat distribution parameters measured with computed tomography (CT)
Yin et al.	2015	China	1073 (43%)	49.43±16.89	HTG	serum triglyceride ≥1000 mg/dL or by visible chylous serum with triglyceride ≥500 mg/dL
Yoon et al.	2017	South Korea	203 (42%)	53.3±17.9	obesity	BMI, abdominal fat distribution parameters measured with computed tomography (CT)
Zhao et al.	2012	China	318 (53%)	diabetic: 57.2±11.0<br>non-diabetic: 44.3±7.8	diabetes	NA

NA: not available, MS: metabolic syndrome, BMI: body-mass index, HT: hypertension, HTG: hypertriglyceridemia, ICD: International Classification of Diseases, NA: not available

Supplementary Figure 1.: Risk of bias assessment

a) **Distribution of risk-of-bias judgements within each bias domain**

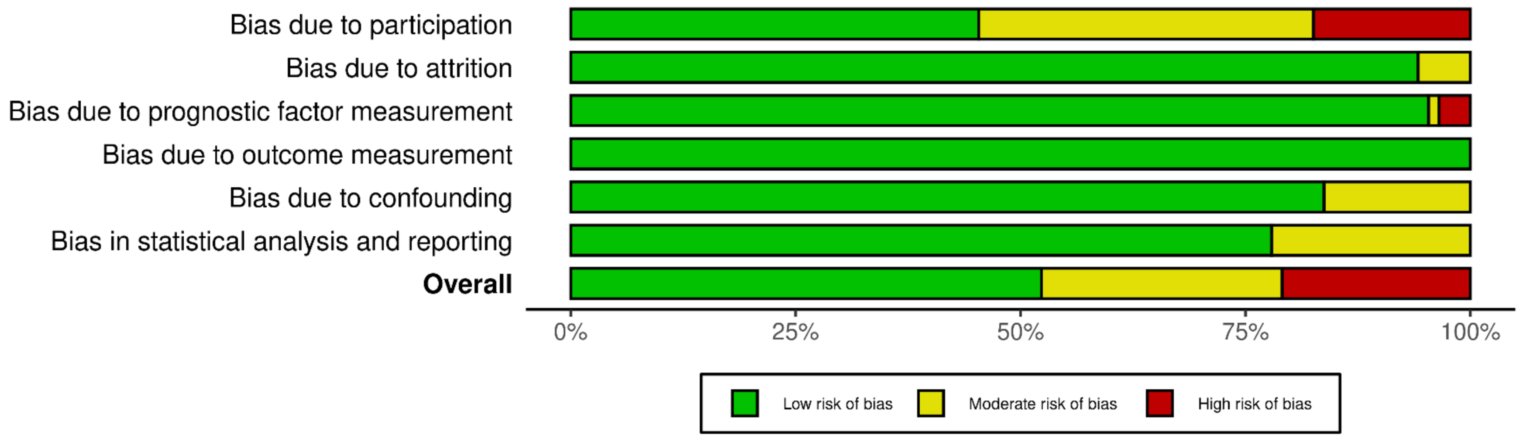

McGuinness, LA, Higgins, JPT. Risk-of-bias VISualization (robvis): An R package and Shiny web app for visualizing risk-of-bias assessments. Res Syn Meth. 2020; 1- 7. [*https://doi.org/10.1002/jrsm.1411*](https://doi.org/10.1002/jrsm.1411)

b) Domain-level judgements for each individual result

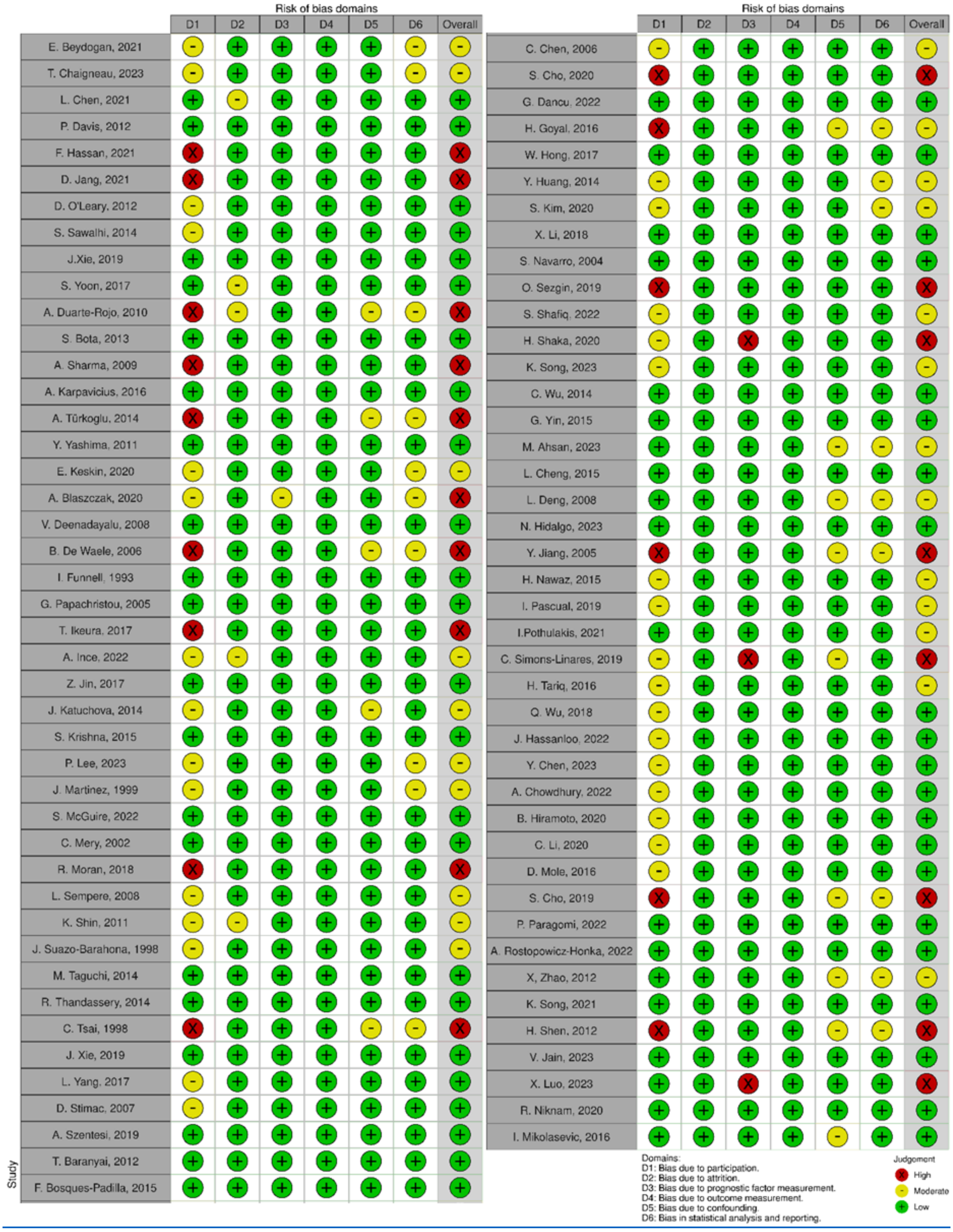

McGuinness, LA, Higgins, JPT. Risk-of-bias VISualization (robvis): An R package and Shiny web app for visualizing risk-of-bias assessments. Res Syn Meth. 2020; 1- 7. https://doi.org/10.1002/jrsm.1411

Supplementary Figure 2.: The odds of developing renal failure with and without excess body weight (EBW)

Study	Sample Size	Obes<br>Obes	Risk	Sample Size	Not Obes<br>Not Obes	Risk	Risk Difference	Definition of<br>Obesity	OR	95%-CI	Weight
L. Yang (2017)	58	5	0.086	38	1	0.026	0.060	≥ 24 kg/m2	3.491	[0.392; 31.117]	9.1%
A. Szentesi (2019)	371	22	0.059	886	12	0.014	0.046	≥ 30 kg/m2	4.591	[2.248; 9.378]	85.6%
J. Suazo-Barahona (1998)	85	8	0.094	65	0	0.000	0.094	≥ 25 kg/m2	14.368	[0.814; 253.677]	5.3%
**Random effect**	**514**	**35**		**989**	**13**				**4.757**	**[2.073; 10.915]**	**100.0%**

I^2 = 0% [0%; 90%], τ = 0

Odds Ratio of Renal Failure

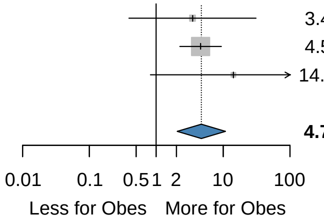

EBW: excess body weight, BMI: body-mass index, OR: odds ratio, CI: confidence interval

Supplementary Figure 3.: The odds of developing respiratory failure with and without EBW

Study	Sample Size	Obes<br>Obes	Risk	Sample Size	Not Obes<br>Not Obes	Risk	Risk Difference	Definition of<br>Obesity	OR	95%-CI	Weight
C. Tsai (1998)	26	3	0.115	294	28	0.095	0.020	≥ 30 kg/m2	1.239	[0.350; 4.388]	0.5%
S. G. Krishna (2015)	52297	4150	0.079	1278005	60008	0.047	0.032	> 40 kg/m2	1.750	[1.693; 1.808]	96.1%
A. Szentesi (2019)	371	27	0.073	886	31	0.035	0.038	≥ 30 kg/m2	2.165	[1.273; 3.681]	2.7%
J. Suazo-Barahona (1998)	85	14	0.165	65	3	0.046	0.119	≥ 25 kg/m2	4.075	[1.119; 14.843]	0.5%
L. Yang (2017)	58	12	0.207	38	2	0.053	0.154	≥ 24 kg/m2	4.696	[0.988; 22.328]	0.3%
**Random effect**	**52837**	**4206**		**1279288**	**60072**				**1.769**	**[1.562; 2.003]**	**100.0%**

I^2 = 2% [0%; 80%], τ = 0.04

Odds Ratio of Respiratory Failure

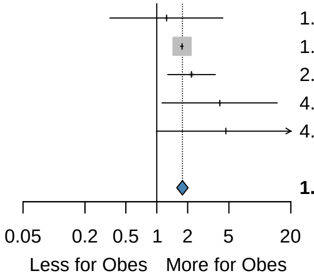

EBW: excess body weight, BMI: body-mass index, OR: odds ratio, CI: confidence interval

Supplementary Figure 4.: The odds of developing moderately severe or severe pancreatitis with and without EBW

Study	Sample Size	with EBW	Risk	Sample Size	without EBW	Risk	Risk Difference	BMI cut-off value	OR	95%-CI	Weight
M. Taguchi (2014)	1396	573	0.410	3767	1511	0.401	0.009	≥ 25 kg/m^2	1.040	[0.917; 1.178]	10.6%
V. Deenadayalu (2008)	30	10	0.333	119	38	0.319	0.014	≥ 30 kg/m2	1.066	[0.455; 2.497]	7.8%
A. Ince (2022)	1032	453	0.439	302	119	0.394	0.045	≥ 25 kg/m2	1.203	[0.926; 1.563]	10.3%
E. Keskin (2020)	77	50	0.649	113	63	0.558	0.092	≥ 30 kg/m2	1.470	[0.809; 2.671]	9.0%
A. Blasszczak (2020)	29412	3166	0.108	29418	1931	0.066	0.042	≥ 40 kg/m2	1.717	[1.619; 1.822]	10.6%
C. M. Mery (2002)	54	19	0.352	34	8	0.235	0.117	≥ 25 kg/m2	1.764	[0.669; 4.652]	7.3%
L. Sempere (2008)	35	13	0.371	50	9	0.180	0.191	≥ 30 kg/m2	2.692	[0.995; 7.283]	7.1%
B. De Waele (2006)	52	16	0.308	186	24	0.129	0.179	≥ 30 kg/m2	3.000	[1.448; 6.215]	8.4%
R. B. Thandassery (2014)	149	125	0.839	131	83	0.634	0.205	≥ 23 kg/m2	3.012	[1.715; 5.290]	9.2%
Z. Jin (2017)	260	52	0.200	342	22	0.064	0.136	≥ 25 kg/m2	3.636	[2.144; 6.167]	9.3%
J. Martinez (1999)	27	11	0.407	22	2	0.091	0.316	≥ 25 kg/m2	6.875	[1.329; 35.577]	4.5%
I. C. Funnell (1993)	19	12	0.632	80	5	0.062	0.569	≥ 30 kg/m2	25.714	[7.012; 94.296]	5.8%
**Random effect**	**32543**	**4500**		**34564**	**3815**				**2.276**	**[1.353; 3.828]**	**100.0%**
**Prediction interval**										**[0.417; 12.430]**	

I^2= 88% [82%; 93%], τ = 0.72

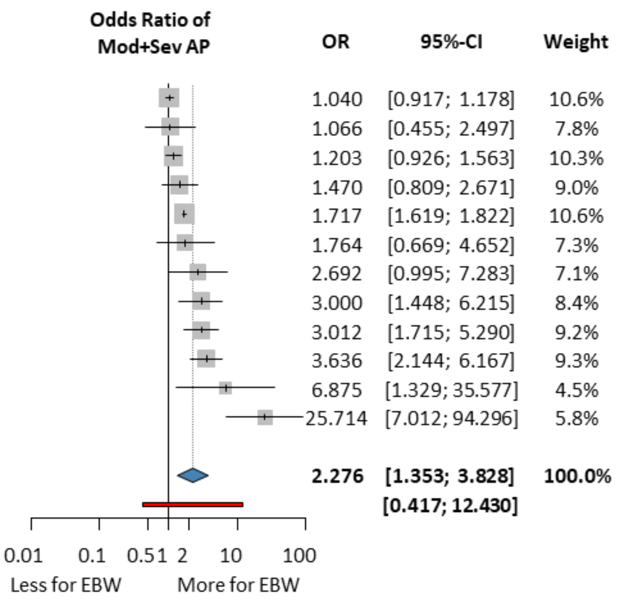

EBW: excess body weight, BMI: body-mass index, Mod: moderately severe, Sev: severe, AP: acute pancreatitis, OR: odds ratio, CI: confidence interval

Supplementary Figure 5.: Funnel plot corresponding to the odds of developing moderately severe or severe pancreatitis with and without EBW**

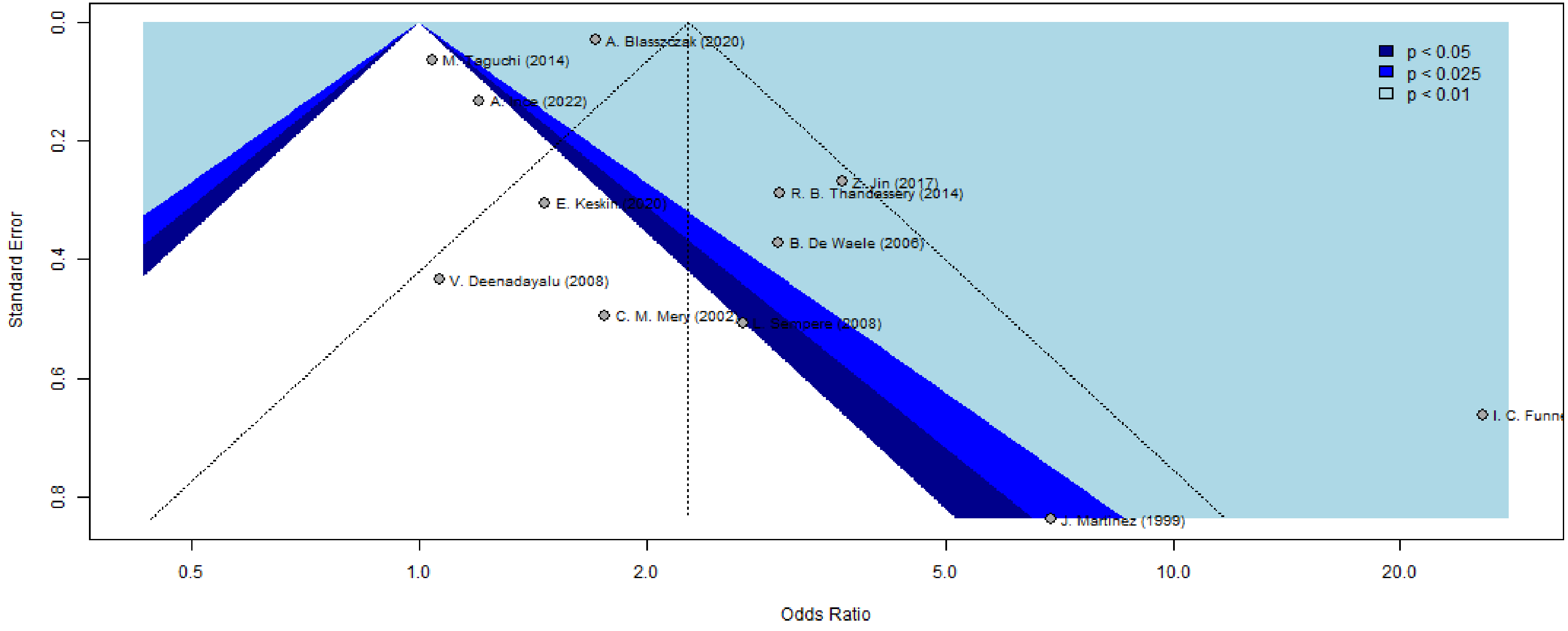

Egger's test (Harbord modification) p-value is 0.3691.

Supplementary Figure 6.: The mean body-mass index (BMI) in the different AP severity groups

Study	Sample Size	Mean [kg/m2]	SD [kg/m2]	Mean [kg/m2]	95%-CI	Weight
**mild pancreatitis**						
Y. Yashima (2011)	76	22.40	3.4000	22.40	[21.64; 23.16]	10.2%
D. Jang (2021)	137	23.30	21.0000	23.30	[19.78; 26.82]	2.7%
S. Yoon (2017)	128	23.40	3.6000	23.40	[22.78; 24.02]	12.3%
J. Xie (2019)	204	24.20	5.0000	24.20	[23.51; 24.89]	13.6%
A. Türkoglu (2014)	62	24.34	3.5200	24.34	[23.46; 25.22]	9.5%
F. Hassan (2021)	56	25.47	0.2100	25.47	[25.41; 25.53]	14.8%
L. Chen (2021) c	70	26.03	3.3971	26.03	[25.23; 26.82]	4.5%
A. Duarte-Rojo (2010) ß	74	26.07	5.2115	26.07	[24.88; 27.26]	7.9%
S. Bota (2013)	207	26.30	4.7000	26.30	[25.66; 26.94]	10.8%
A. Sharma (2009)	108	26.90	7.1000	26.90	[25.56; 28.24]	6.7%
S. Sawalhi (2014)	124	30.00	7.1000	30.00	[28.75; 31.25]	7.0%
**Random effect**	**1246**			**25.47**	**[24.12; 26.81]**	**100.0%**
**moderately severe pancreatitis**						
J. Xie (2019)	56	24.70	2.7000	24.70	[23.99; 25.41]	34.1%
S. Yoon (2017)	62	25.70	4.1000	25.70	[24.68; 26.72]	29.1%
F. Hassan (2021)	15	29.54	0.6200	29.54	[29.23; 29.85]	36.8%
**Random effect**	**133**			**27.70**	**[25.84; 29.56]**	**100.0%**
**severe pancreatitis**						
S. Yoon (2017)	13	24.20	4.4000	24.20	[21.81; 26.59]	8.5%
Y. Yashima (2011)	48	24.40	4.3000	24.40	[23.18; 25.62]	11.4%
J. Xie (2019)	46	25.30	3.1000	25.30	[24.40; 26.20]	15.3%
A. Duarte-Rojo (2010) ß	25	26.86	5.4945	26.86	[24.70; 29.01]	8.0%
T. Chaigneau (2023)	65	28.10	5.7000	28.10	[26.71; 29.49]	4.9%
S. Bota (2013)	127	28.10	5.7000	28.10	[27.11; 29.09]	12.3%
A. Türkoglu (2014)	30	29.67	4.1600	29.67	[28.18; 31.16]	10.4%
F. Hassan (2021)	9	29.87	1.6700	29.87	[28.78; 30.96]	14.8%
A. Sharma (2009)	25	30.70	7.8000	30.70	[27.64; 33.76]	5.7%
S. Sawalhi (2014)	16	31.00	5.9000	31.00	[28.11; 33.89]	6.1%
A. Karpavicius (2016)	20	31.07	10.0200	31.07	[26.68; 35.46]	2.6%
**Random effect**	**424**			**27.94**	**[26.52; 29.37]**	**100.0%**

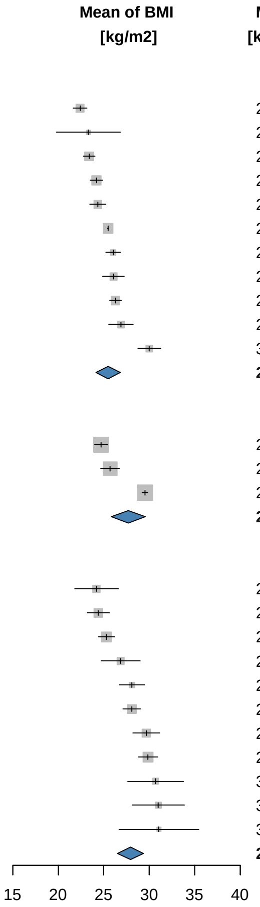

Total I^2 = 98% [94%; 99%]

Between study I2: 78% [74%; 77%] Within study I2:20% [19%; 22%]

BMI: body-mass index, CI: confidence interval p= 0.0004

Supplementary Figure 7.: Funnel plot corresponding to the mean body-mass index (BMI) in the different AP severity groups

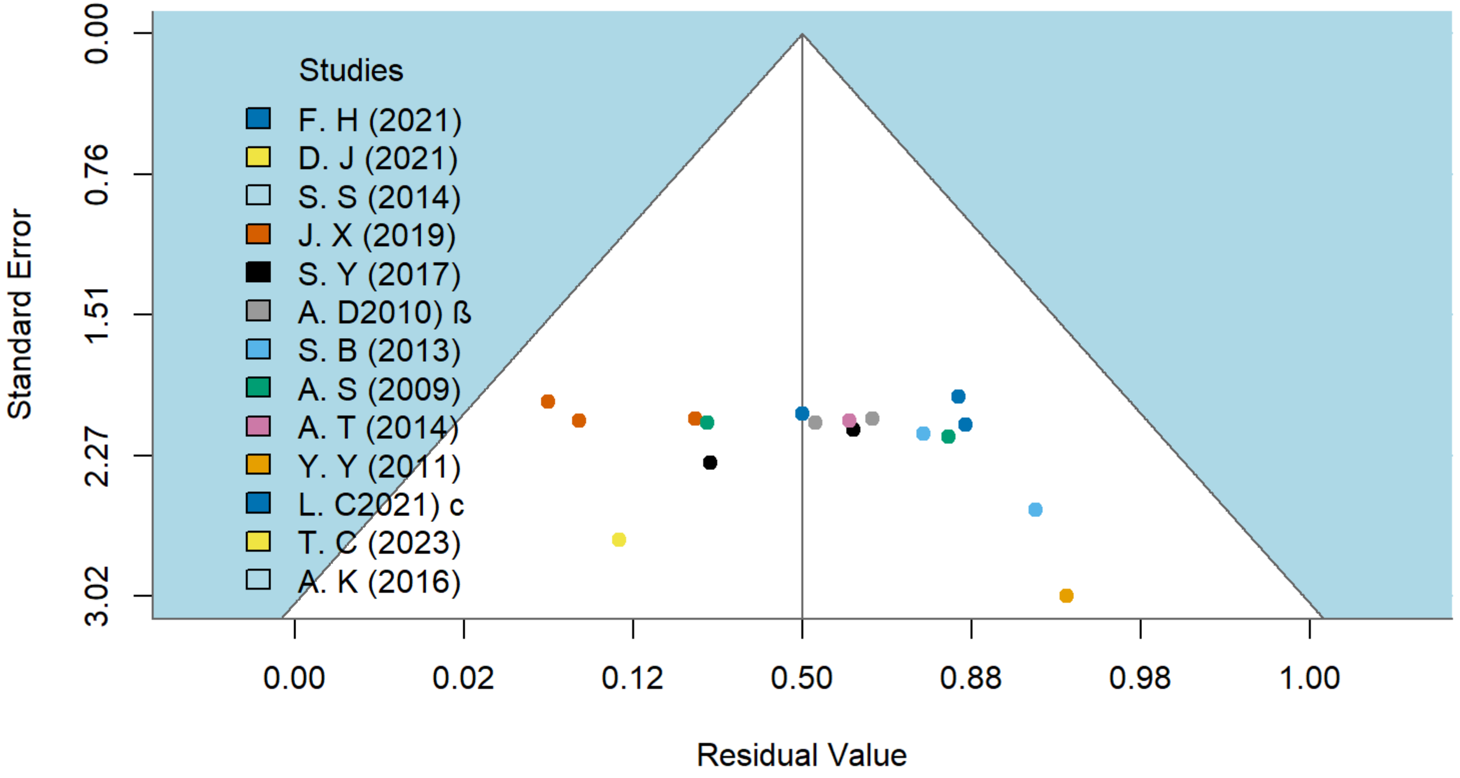

Supplementary Figure 8.: The mean volume of subcutaneous adipose tissue (SAT) in the different AP severity groups

Study	Sample Size	Mean [cm2]	SD [cm2]	Mean [cm2]	95%-CI	Weight
**mild pancreatitis**						
Y. Yashima (2011)	76	98.80	6.2000	98.80	[97.41; 100.19]	23.8%
S. Yoon (2017)	128	116.00	61.0000	116.00	[105.43; 126.57]	18.4%
D. Jang (2021)	137	119.70	70.6000	119.70	[107.88; 131.52]	17.3%
L. Chen (2021) c	70	126.49	52.4705	126.49	[114.19; 138.78]	16.9%
J. Xie (2019)	204	128.10	22.1000	128.10	[125.07; 131.13]	23.5%
**Random effect**	**615**			**117.18**	**[105.68; 128.67]**	**100.0%**
**moderately severe pancreatitis**						
J. Xie (2019)	56	138.80	22.4000	138.80	[132.93; 144.67]	67.2%
S. Yoon (2017)	62	148.00	86.0000	148.00	[126.59; 169.41]	32.8%
**Random effect**	**118**			**140.98**	**[120.96; 161.00]**	**100.0%**
**severe pancreatitis**						
S. Yoon (2017)	13	124.00	43.0000	124.00	[100.63; 147.37]	18.0%
Y. Yashima (2011)	48	128.40	8.9000	128.40	[125.88; 130.92]	43.3%
J. Xie (2019)	46	134.60	25.8000	134.60	[127.14; 142.06]	38.7%
**Random effect**	**107**			**130.50**	**[114.91; 146.10]**	**100.0%**

Mean of SAT [cm2]

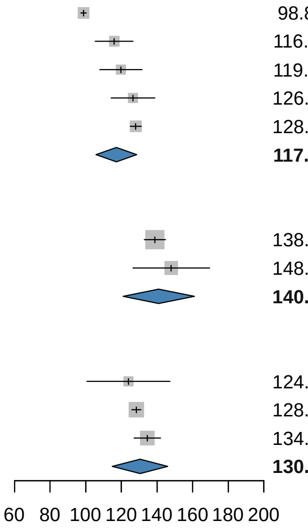

Total I^2 = 95% [77%; 99%]

Between study I2: 11% [0%; 50%] Within study I2:85% [77%; 50%]

Omnibus test for subgroup difference: p= 0.0831

SAT: subcutaneous adipose tissue, CI: confidence interval

Supplementary Figure 9.: The mean volume of visceral adipose tissue (VAT) in the different AP severity groups

Study	Sample Size	Mean [cm2]	SD [cm2]	Mean [cm2]	95%-CI	Weight
**mild pancreatitis**						
Y. Yashima (2011)	76	102.20	6.0900	102.20	[100.83; 103.57]	36.3%
S. Yoon (2017)	128	105.00	70.0000	105.00	[92.87; 117.13]	13.9%
J. Xie (2019)	204	121.60	17.3000	121.60	[119.23; 123.97]	45.7%
D. Jang (2021)	137	123.90	67.7000	123.90	[112.56; 135.24]	2.1%
L. Chen (2021) c	70	176.13	67.3867	176.13	[160.34; 191.91]	2.0%
**Random effect**	**615**			**125.91**	**[95.67; 156.15]**	**100.0%**
**moderately severe pancreatitis**						
S. Yoon (2017)	62	150.00	59.0000	150.00	[135.31; 164.69]	23.4%
J. Xie (2019)	56	154.10	15.1000	154.10	[150.15; 158.05]	76.6%
**Random effect**	**118**			**158.98**	**[126.90; 191.07]**	**100.0%**
**severe pancreatitis**						
Y. Yashima (2011)	48	149.50	10.0000	149.50	[146.67; 152.33]	44.2%
J. Xie (2019)	46	178.70	15.2000	178.70	[174.31; 183.09]	52.0%
S. Yoon (2017)	13	184.00	81.0000	184.00	[139.97; 228.03]	3.8%
**Random effect**	**107**			**179.07**	**[147.70; 210.44]**	**100.0%**

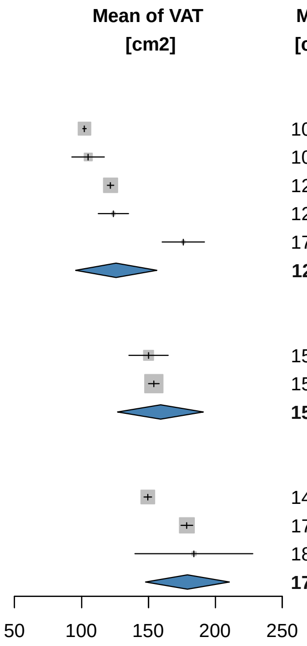

Total I^2 = 100% [28%; 100%]

Between study I2: 97% [0%; 87%] Within study I2:3% [28%; 13%]

Omnibus test for subgroup difference: p= <0.0001

VAT: visceral adipose tissue, CI: confidence interval

Supplementary Figure 10.: The odds of mortality with and without diabetes

Study	Diabetes Sample Size	Diabetes Event	Diabetes Risk	No Diabetes Sample Size	No Diabetes Event	No Diabetes Risk	Risk Difference	OR	95%-CI	Weight
C. Li (2020)	109	4	0.037	633	29	0.046	-0.009	0.793	[0.273; 2.303]	26.0%
Y. Chen (2023)	29	8	0.276	120	23	0.192	0.084	1.607	[0.632; 4.083]	27.2%
A. Rostopowicz-Honka (2022)	68	4	0.059	265	8	0.030	0.029	2.008	[0.586; 6.876]	24.4%
X. Zhao (2012)	40	6	0.150	278	3	0.011	0.139	16.176	[3.867; 67.662]	22.5%
**Random effect**	**246**	**22**		**1296**	**63**			**2.373**	**[0.320; 17.570]**	**100.0%**

I^2 = 73% [25%; 91%], τ = 1.11

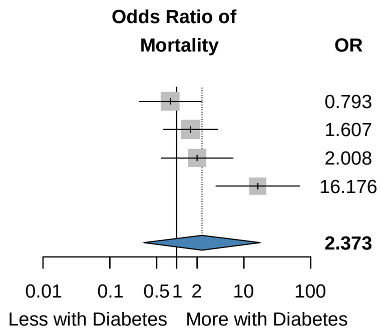

OR: odds ratio, CI: confidence interval

Supplementary Figure 11.: The odds of respiratory failure with and without diabetes

Study	Diabetes Sample Size	Diabetes Event	Diabetes Risk	No Diabetes Sample Size	No Diabetes Event	No Diabetes Risk	Risk Difference	OR	95%-CI	Weight
H. Nawaz (2015)	1349	41	0.030	6050	177	0.029	0.001	1.040	[0.737; 1.468]	29.2%
H. Shen (2012)	18990	1177	0.062	37980	2165	0.057	0.005	1.093	[1.016; 1.176]	56.7%
A. Szentesi (2019)	206	15	0.073	1051	43	0.041	0.032	1.841	[1.003; 3.381]	14.1%
**Random effect**	**20545**	**1233**		**45081**	**2385**			**1.159**	**[0.653; 2.058]**	**100.0%**

I^2 = 31% [0%; 93%], τ = 0.17

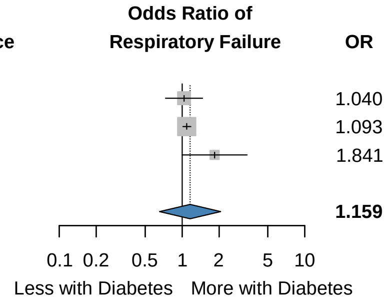

OR: odds ratio, CI: confidence interval

frontiers

Supplementary Figure 12.: The odds of developing severe AP with and without HTG etiology

Study	High Triglicieride Sample Size	High Triglicieride Event	High Triglicieride Risk	Normal Triglicieride Sample Size	Normal Triglicieride Event	Normal Triglicieride Risk	Risk Difference	OR	95%-CI	Weight
G. Yin (2015)	208	11	0.053	865	35	0.040	0.012	1.324	[0.661; 2.653]	15.4%
S. Cho (2020)	30	3	0.100	293	21	0.072	0.028	1.439	[0.403; 5.139]	7.9%
G. Dancu (2022)	30	5	0.167	232	27	0.116	0.050	1.519	[0.536; 4.299]	10.3%
Y. Huang (2014)	123	21	0.171	828	72	0.087	0.084	2.162	[1.275; 3.666]	18.6%
W. Hong (2017)	36	7	0.194	611	51	0.083	0.111	2.650	[1.106; 6.350]	12.5%
S. Kim (2020)	52	5	0.096	156	6	0.038	0.058	2.660	[0.776; 9.110]	8.3%
C. Chen (2006)	4	4	1.000	72	47	0.653	0.347	4.832	[0.250; 93.342]	1.9%
T. Baranyai (2012)	26	13	0.500	388	55	0.142	0.358	6.055	[2.667; 13.747]	13.3%
H. Goyal (2016)	30	13	0.433	147	13	0.088	0.345	7.882	[3.143; 19.771]	11.8%
**Random effect**	**539**	**82**		**3592**	**327**			**2.651**	**[1.602; 4.387]**	**100.0%**
**Prediction interval**									**[0.851; 8.259]**	

I^2 = 49% [0%; 76%], τ = 0.43

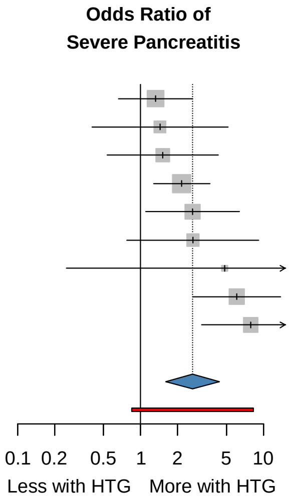

HTG: hypertriglyceridemia, OR: odds ratio, CI: confidence interval

Supplementary Figure 13.: The odds of developing necrosis with and without HTG etiology

Study	High Trigliceride Sample Size	High Trigliceride Event	High Trigliceride Risk	Normal Trigliceride Sample Size	Normal Trigliceride Event	Normal Trigliceride Risk	Risk Difference	OR	95%-CI	Weight
X. Li (2018)	305	3	0.010	425	5	0.012	-0.002	0.834	[0.198; 3.518]	5.6%
X. Li (2018)	305	213	0.698	425	292	0.687	0.011	1.055	[0.766; 1.451]	26.8%
S. Kim (2020)	52	8	0.154	156	18	0.115	0.038	1.394	[0.567; 3.426]	11.4%
K. Song (2023)	285	69	0.242	587	81	0.138	0.104	1.996	[1.394; 2.856]	25.5%
K. Song (2023)	285	24	0.084	587	24	0.041	0.043	2.157	[1.202; 3.870]	18.4%
T. Baranyai (2012)	26	7	0.269	388	34	0.088	0.182	3.836	[1.505; 9.774]	10.8%
S. Navarro (2004)	19	3	0.158	19	0	0.000	0.158	8.273	[0.398; 172.054]	1.5%
**Random effect**	**1277**	**327**		**2587**	**454**			**1.709**	**[1.070; 2.728]**	**100.0%**

I^2 = 58% [3%; 82%], τ = 0.33

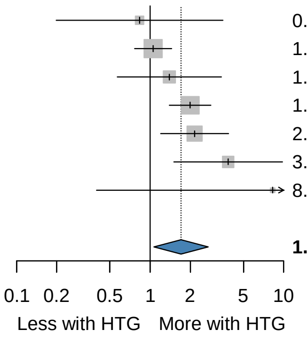

HTG: hypertriglyceridemia, OR: odds ratio, CI: confidence interval

Supplementary Figure 14.: The odds of developing renal failure with and without HTG etiology

Study	High Trigliceride Sample Size	High Trigliceride Event	High Trigliceride Risk	Normal Trigliceride Sample Size	Normal Trigliceride Event	Normal Trigliceride Risk	Risk Difference	OR	95%-CI	Weight
G. Yin (2015)	208	7	0.034	865	20	0.023	0.011	1.471	[0.614; 3.527]	26.8%
K. Song (2023)	285	42	0.147	587	56	0.095	0.052	1.639	[1.068; 2.514]	31.5%
S. Kim (2020)	52	3	0.058	156	4	0.026	0.032	2.327	[0.503; 10.757]	19.0%
Y. Huang (2014)	123	8	0.065	828	4	0.005	0.060	14.330	[4.248; 48.345]	22.6%
**Random effect**	**668**	**60**		**2436**	**84**			**2.780**	**[0.531; 14.547]**	**100.0%**

I^2 = 74% [26%; 91%], τ = 0.90

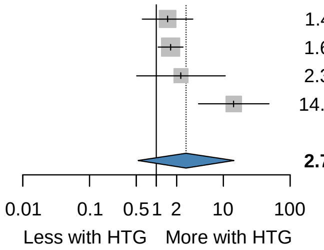

HTG: hypertriglyceridemia, OR: odds ratio, CI: confidence interval

Supplementary Figure 15.: The odds of falling into septic shock with and without HTG etiology

Study	HTG Sample Size	HTG Event	No HTG Sample Size	No HTG Event	Raw data OR	Odds Ratio of Schock	OR	95% CI	Weight	Adjusted for
H. Shaka (2020)	104465	418	470765	3295	0.57		0.64	[0.48; 0.85]	23.9%	age, sex, race, comorbidity
X. Li (2018)	305	49	425	66	1.04		1.04	[0.70; 1.56]	23.3%	
K. Song (2023)	285	29	587	30	2.10		2.10	[1.24; 3.58]	22.4%	
S. Navarro (2004)	19	1	19	0	3.16		3.16	[0.12; 82.64]	5.4%	
Y. Huang (2014)	123	5	828	5	6.97		6.97	[1.99; 24.45]	16.1%	
S. Kim (2020)	52	3	156	1	9.49		9.49	[0.96; 93.32]	8.9%	
**Random effect**							**1.91**	**[0.65; 5.62]**	**100.0%**	

I^2 = 84% [65%; 92%], τ = 0.88

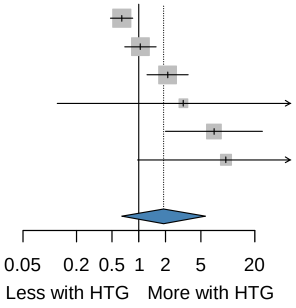

HTG: hypertriglyceridemia, OR: odds ratio, CI: confidence interval

Supplementary Figure 16.: The odds of mortality in AP with and without HTG etiology

Study	High Trigliceride			Normal Trigliceride			Risk Difference	Odds Ratio of Mortality	OR	95%-CI	Weight
	Sample Size	Event	Risk	Sample Size	Event	Risk					
S. Shafiq (2022)	21	0	0.000	529	61	0.115	-0.115		0.177	[0.011; 2.962]	4.0%
G. Yin (2015)	208	1	0.005	865	22	0.025	-0.021		0.185	[0.025; 1.381]	6.9%
G. Dancu (2022)	30	1	0.033	232	22	0.095	-0.061		0.329	[0.043; 2.535]	6.7%
H. Shaka (2020)	104465	627	0.006	470765	3766	0.008	-0.002		0.749	[0.688; 0.815]	25.8%
X. Li (2018)	305	24	0.079	425	36	0.085	-0.006		0.923	[0.538; 1.582]	21.7%
T. Baranyai (2012)	26	0	0.000	388	7	0.018	-0.018		0.960	[0.053; 17.263]	3.9%
S. Navarro (2004)	19	0	0.000	19	0	0.000	0.000		1.000	[0.019; 52.977]	2.2%
O. Sezgin (2019)	33	1	0.030	602	15	0.025	0.005		1.223	[0.157; 9.550]	6.7%
Y. Huang (2014)	123	5	0.041	828	11	0.013	0.027		3.147	[1.075; 9.217]	14.5%
H. Goyal (2016)	30	1	0.033	147	1	0.007	0.027		5.034	[0.306; 82.815]	4.1%
S. Kim (2020)	52	2	0.038	156	0	0.000	0.038		15.495	[0.732; 328.129]	3.5%
**Random effect**	**105312**	**662**		**474956**	**3941**				**0.987**	**[0.490; 1.988]**	**100.0%**
**Prediction interval**										**[0.206; 4.725]**	

I^2 = 40% [0%; 70%], τ = 0.62

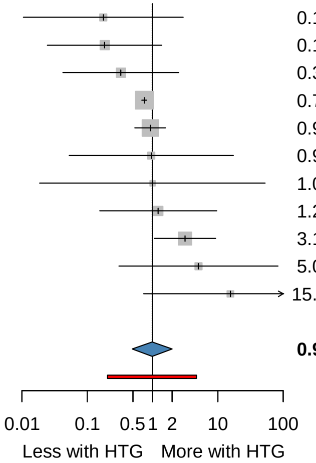

HTG: hypertriglyceridemia, OR: odds ratio, CI: confidence interval

Supplementary Figure 17.: Funnel plot corresponding to the odds of mortality in AP with and without HTG etiology

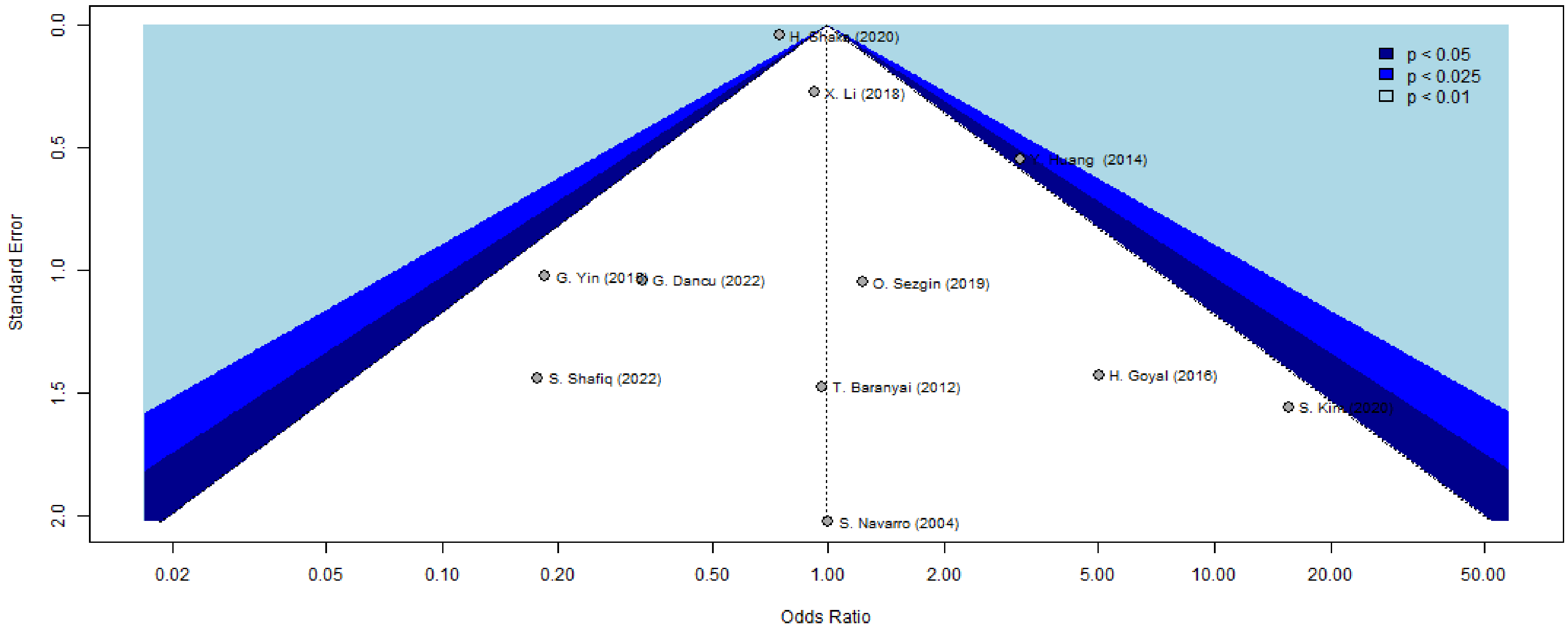

Egger's test (Harbord modification) p-value is 0.4215.

Supplementary Figure 18.: The odds of mortality in the groups of patients with and without metabolic syndrome (MS)

Study	MS Sample Size	MS Event	MS Risk	No MS Sample Size	No MS Event	No MS Risk	Risk Difference	OR	95%-CI	Weight
A. Szentesi (2019)	167	2	0.012	739	16	0.022	-0.010	0.548	[0.125; 2.405]	20.8%
A.M. Blaszczak (2020)	136712	1154	0.008	137575	1875	0.014	-0.005	0.616	[0.572; 0.663]	35.8%
R. Niknam (2020)	34	2	0.059	42	1	0.024	0.035	2.562	[0.222; 29.534]	12.1%
I. Mikolasevic (2016)	110	18	0.164	499	22	0.044	0.120	4.242	[2.189; 8.221]	31.3%
**Random effect**	**137023**	**1176**		**138855**	**1914**			**1.307**	**[0.240; 7.102]**	**100.0%**

I^2 = 91% [80%; 96%], τ = 0.89

Odds Ratio of Mortality

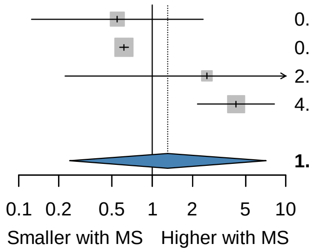

MS: metabolic syndrome, OR: odds ratio, CI: confidence interval

References

1. Mantel, N. and W. Haenszel, *Statistical Aspects of the Analysis of Data From Retrospective Studies of Disease.* JNCI: Journal of the National Cancer Institute, 1959. **22**(4): p. 719-748.
2. Robins, J., S. Greenland, and N.E. Breslow, *A general estimator for the variance of the Mantel-Haenszel odds ratio.* Am J Epidemiol, 1986. **124**(5): p. 719-23.
3. *The Handbook of Research Synthesis and Meta-Analysis*, ed. H. Cooper, L.V. Hedges, and J.C. Valentine. 2019: Russell Sage Foundation.
4. Sweeting, M.J., A.J. Sutton, and P.C. Lambert, *What to add to nothing? Use and avoidance of continuity corrections in meta-analysis of sparse data.* Stat Med, 2004. **23**(9): p. 1351-75.
5. Knapp, G. and J. Hartung, *Improved tests for a random effects meta-regression with a single covariate.* Stat Med, 2003. **22**(17): p. 2693-710.
6. IntHout, J., J.P. Ioannidis, and G.F. Borm, *The Hartung-Knapp-Sidik-Jonkman method for random effects meta-analysis is straightforward and considerably outperforms the standard DerSimonian-Laird method.* BMC Med Res Methodol, 2014. **14**: p. 25.
7. Jackson, D., et al., *The Hartung-Knapp modification for random-effects meta-analysis: A useful refinement but are there any residual concerns?* Stat Med, 2017. **36**(25): p. 3923-3934.
8. Paule, R.C. and J. Mandel, *Consensus Values and Weighting Factors.* J Res Natl Bur Stand (1977), 1982. **87**(5): p. 377-385.
9. Harrer, M., et al., *Doing Meta-Analysis with R: A Hands-On Guide*. 2021.
10. Veroniki, A.A., et al., *Methods to estimate the between-study variance and its uncertainty in meta-analysis.* Res Synth Methods, 2016. **7**(1): p. 55-79.
11. Cheung, M.W., *Modeling dependent effect sizes with three-level meta-analyses: a structural equation modeling approach.* Psychol Methods, 2014. **19**(2): p. 211-29.
12. Harbord, R.M., R.J. Harris, and J.A.C. Sterne, *Updated Tests for Small-study Effects in Meta-analyses.* The Stata Journal, 2009. **9**(2): p. 197-210.
